# Key Inflammatory Pathways, Biomarkers, and Targeted Management Strategies in Primary Total Joint Arthroplasty: A Narrative Review

**DOI:** 10.3390/medicina62091713

**Published:** 2026-09-06

**Authors:** Adelina-Elena Moise, Mihai Emanuel Gherghe, Alex-Gabriel Grigore, Iosif-Aliodor Timofticiuc, Matei Todor, Patricia Balaban, Constantin-Adrian Andrei, Serban Dragosloveanu, Constantin Caruntu, Cristian Scheau

**Affiliations:** 1 Faculty of Medicine, The “Carol Davila” University of Medicine and Pharmacy, 050474 Bucharest, Romania; adelina-elena.moise@drd.umfcd.ro (A.-E.M.); mihai-emanuel.gherghe@drd.umfcd.ro (M.E.G.); iosif-aliodor.timofticiuc2022@stud.umfcd.ro (I.-A.T.); matei.todor2022@stud.umfcd.ro (M.T.); patricia.balaban2022@stud.umfcd.ro (P.B.); constantin-adrian.andrei@drd.umfcd.ro (C.-A.A.); constantin.caruntu@umfcd.ro (C.C.); cristian.scheau@umfcd.ro (C.S.); 2 “Foisor” Clinical Hospital of Orthopaedics, Traumatology and Osteoarticular TB, 021382 Bucharest, Romania; alexgrigore01@gmail.com; 3 Department of Dermatology, “Prof. N.C. Paulescu” National Institute of Diabetes, Nutrition and Metabolic Diseases, 011233 Bucharest, Romania

**Keywords:** total joint arthroplasty, perioperative inflammation, DAMPs, periprosthetic joint infection, biomarkers, cytokines, osteoimmunology, aseptic loosening, clinical decision-making

## Abstract

Total joint arthroplasty is a surgical procedure with rising global incidence. Although a strong postoperative inflammatory response is necessary for tissue repair following primary arthroplasty, it may prove to be harmful if excessive or prolonged. This could compromise osseointegration, increase pain, and delay the detection of periprosthetic joint infection. This narrative review examines the principal inflammatory pathways activated by primary arthroplasty. Damage-associated molecular patterns produced by injury and cell death, such as High Mobility Group Box 1 Protein, cell-free DNA, extracellular ATP, histones, and heat shock proteins, trigger innate immune activation following surgical trauma. These mediators use inflammasome pathways and pattern recognition receptors to intensify inflammatory signaling. The acute-phase trajectory, characterized by increases in C-reactive protein and erythrocyte sedimentation rate, alongside the role of interleukin-6 as a precursor factor, is examined together with synovial markers to facilitate the differentiation between septic and aseptic inflammation. Cytokine signaling cascades (JAK-STAT, NF-κB, MAPK) and the RANK/RANKL/OPG axis at the bone-immune interface are also considered. This manuscript highlights relevant inflammatory pathways, clinically significant biomarkers, and pathway-guided management strategies to provide an overview of the current research on the biological mechanisms underlying perioperative inflammation in primary arthroplasty. No inflammatory biomarker has yet been validated as a predictor of aseptic loosening.

## 1. Introduction

Total joint arthroplasty initiates a robust postoperative inflammatory response as part of normal tissue healing [1]. Inflammation is a protective mechanism against trauma, the presence of foreign bodies, or irritation and imbalance caused by toxins, which allows protective circulating elements to reach the affected site [2]. Surgical procedures such as bone cutting, reaming, and soft tissue dissection during surgery cause immediate cellular injury and trigger the release of pro-inflammatory mediators [3,4]. Systemic inflammatory cytokines and acute-phase reactants, including C-reactive protein (CRP), increase in the bloodstream within hours [5,6,7,8]. Although postoperative inflammation plays a role in protecting and healing affected areas, it is often necessary to control this process to prevent the induction of unbalanced catabolism that can seriously affect healing [9]. Over a longer time frame, a sustained inflammatory response to wear debris contributes to periprosthetic osteolysis and late implant failure [10,11]. Postoperative inflammation can also mimic infection, complicating early diagnosis of periprosthetic joint infection (PJI) [12,13]. Differentiating normal postoperative inflammation from early infection or other conditions can be challenging and may result in delayed treatment or unnecessary interventions [14]. This difficulty is compounded when antimicrobial treatment is started before adequate sampling, which reduces culture yield and can obscure diagnosis [15].

Although inflammation significantly affects arthroplasty outcomes, approaches to postoperative inflammation management vary widely among surgical centers and clinicians. There is no universally standardized protocol; practices differ based on resources, clinical culture, and evolving evidence [16,17,18,19]. For instance, while non-steroidal anti-inflammatory drugs (NSAIDs) are commonly used in multimodal analgesia [20], specific regimens differ between institutions [21,22,23,24]. Many anesthesiologists now administer a single low-dose dexamethasone during joint replacement, as evidence shows it can reduce postoperative inflammation, pain, and nausea, and may shorten hospital stays without increasing infection risk [25,26,27]. Along with this invasive action, multiple inflammatory pathways are stimulated, which in turn generate pain and local and systemic inflammatory responses, with possible risks of infections [28,29]. In general, inflammation in this type of surgery arises from a synergistic effect of multiple pathways, such as the innate immune pathway [28], cytokine and chemokine signaling [30], bone-remodeling inflammation [31,32], and infection-driven inflammation caused directly by pathogens [33].

Taken together, postoperative inflammation following primary arthroplasty should be considered a multifactorial, tightly regulated biological process rather than the effect of a single mediator [34]. The same multiplicity characterizes the later response to wear debris, in which several pathways act in parallel [35,36]. Consequently, the substantial heterogeneity observed in contemporary inflammation-management strategies reflects, at least in part, an incomplete understanding of the underlying physiological and immunological processes that govern normal versus pathological inflammatory responses after joint replacement surgery [22,24,34]. To reach consensus on postoperative inflammation management, a mechanistic, pathway-based framework is necessary. This approach should clarify how surgical trauma activates specific inflammatory cascades, how these pathways interact locally and systemically, and how their biomarkers relate to clinical outcomes and complications [37,38,39,40]. A comprehensive understanding of these processes and their timing is essential for developing targeted, evidence-based strategies that modulate inflammation while preserving its role in tissue repair and implant integration [41,42].

Building on this need, the present review aims to answer a single question: how the molecular events initiated by surgical trauma (and, when present, by bacterial colonization) manifest as measurable local and systemic signals, and how these signals should be interpreted in the perioperative decision-making after primary total joint arthroplasty. To address it, every pathway discussed in this paper is presented in the same format: the initiating trigger, the signaling cascade it activates, the biomarkers that report on that cascade, the clinical outcome resulting from the biomarkers in question, and the interventions that are clinically available to modulate it. Section 3, Section 4, Section 5 and Section 6 follow this structure, so that mechanistic detail is presented in synergy with its clinical outcome. The scope is restricted to primary total arthroplasties and to the interval extending from the immediate perioperative period to the first postoperative year, during which both sterile and infection-driven inflammatory responses determine implant integration and early complication risk. Topics that intersect with this question without being central to it are treated selectively: biomaterial design, three-dimensional bioprinting and regenerative strategies are considered only if they modulate the host inflammatory response, and are not reviewed comprehensively. Revision arthroplasty, non-articular implants and pediatric populations are not included in the scope of this review. What distinguishes this review from existing papers on postoperative inflammation is: firstly, the level of evidence supporting each mechanism and each therapeutic strategy is stated comprehensively, so that findings regarding clinical studies are differentiated from those obtained in vivo (animal models) or in vitro; secondly, biomarker data are integrated into a practical framework for distinguishing the expected postoperative inflammatory response from periprosthetic joint infection, and for linking that distinction to subsequent management.

## 2. Literature Search Strategy

This work was conducted as a narrative review. It does not follow a registered systematic review protocol, and no formal risk-of-bias assessment was performed. The literature was updated to include relevant studies, reviews, and guidelines published up to 2026. Relevant publications were identified through searches in major biomedical databases, and search terms included combinations of keywords related to periprosthetic joint infection, inflammatory biomarkers, diagnostic accuracy, damage-associated molecular patterns, cytokine and chemokine signaling, osteoimmunology and macrophage polarization, adaptive immune-bone interactions, osteoblasts–osteoclasts coupling, aseptic loosening and implant survivorship, and perioperative anti-inflammatory management. Reference lists of retrieved articles were screened manually for further relevant publications. Only English-language articles were considered. Conference abstracts, editorials, books and letters without original data were excluded. Articles were selected by consensus among the authors on the basis of conceptual relevance rather than predefined quantitative screening criteria.

## 3. Innate Immune System and Acute-Phase Response Pathways

The direct and immediate effect of any surgical intervention is local trauma. In the case of total arthroplasty, the damage can be caused by several factors: the direct effect of the cutter, the temperature used in the electrocautery, the temperature produced by the exothermic reactions of the cements used in these types of interventions, or ischemia produced by the tourniquet [43,44,45,46]. All of these factors can directly trigger the formation of damage-associated molecular patterns (DAMPs) [47], and can also activate acute-phase response, which follows a predictable trajectory after surgical trauma [3,34]. Comparable kinetic profiles have been described after two-stage exchange for periprosthetic joint infection [48].

### 3.1. DAMP-Based Pro-Inflammatory Pathway

#### 3.1.1. Mechanistic Overview

The main trigger for DAMP formation is cell death, which can occur by apoptosis, necroptosis, pyroptosis, or even ferroptosis [47]. Many types of cells, such as neutrophils, dendritic cells, monocytes, epithelial cells, endothelial cells, fibroblasts, etc., act as a source of DAMPs, which can include: High Mobility Group Box 1 Protein (HMGB1), cell-free DNA, adenosine triphosphate (ATP), histones, and heat shock proteins (HSP) [49].

Besides its release after cellular death, HMGB1 can also be secreted by cells that control bone metabolism, such as osteoblasts, osteoclasts, and chondrocytes, directly promoting the migration of osteoblasts and mesenchymal stem cells, facilitating their differentiation along the osteoblastic pathway [50]. Cell-free deoxyribonucleic acid (cf-DNA), another key DAMP, which is found in synovial fluids in patients with joint-related disease, can bind to various inflammatory markers, such as other DAMPs, HMGB, or even immunoglobulin complexes, leading to cascade activation of the innate immune system and also an overexpression of cytokines [51]. ATP is often referred to as the ubiquitous energy currency, and it fuels almost all the key metabolic pathways of the cells [52]. Together with the emergence of the purinergic theory and vast measurements of the ATP levels in vivo, it was also discovered that ATP molecules are found in large quantities intracellularly, and with the massive efflux caused by cell death or cellular trauma, pro-inflammatory mechanisms are activated [53]. While histones and heat shock proteins are often referred to in articles as DAMPs [47,54,55,56,57,58,59,60], they are not adequately reported in original research discussing the inflammation in total primary arthroplasty.

**Figure 1:**
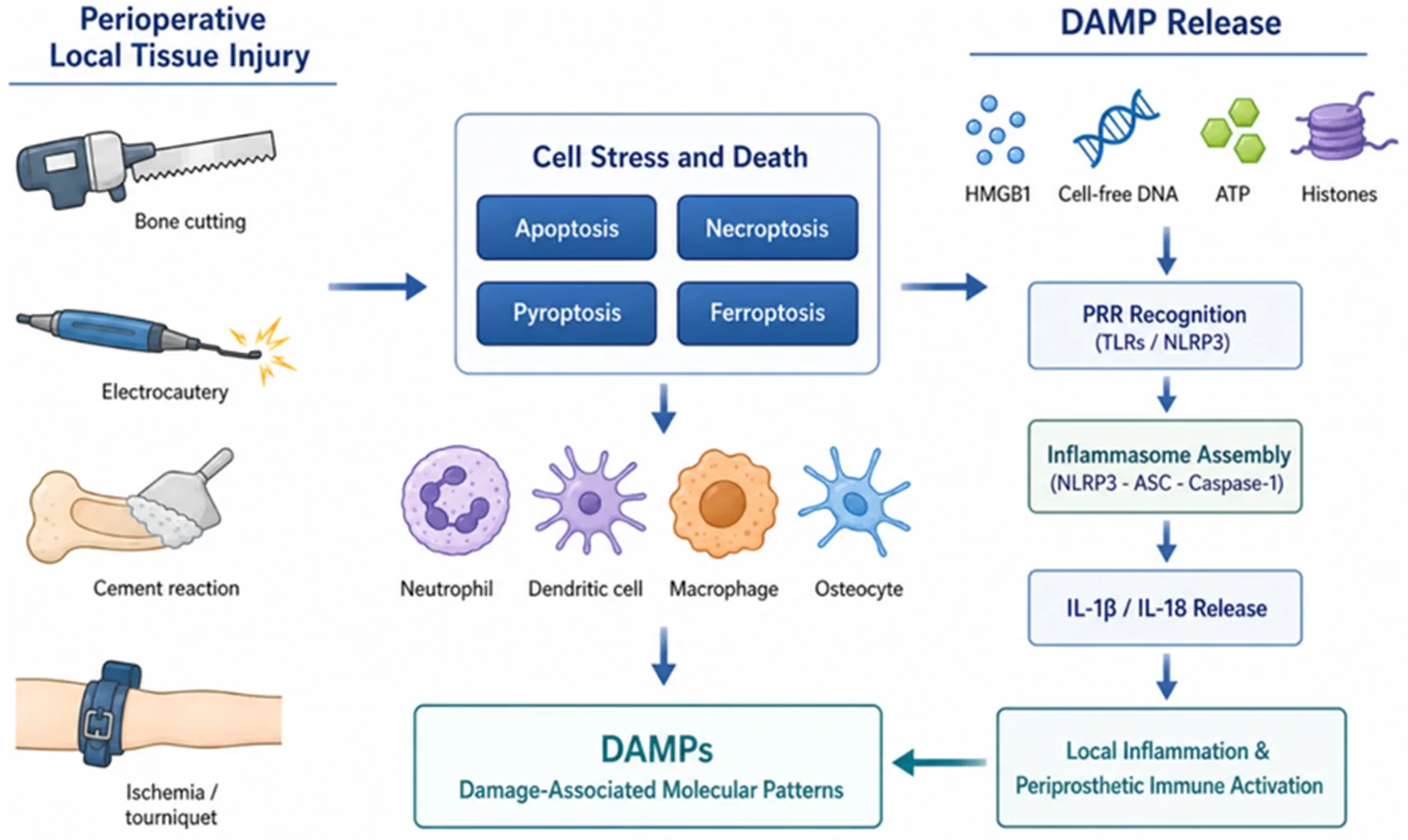
Mechanistic pathway linking surgical trauma during total arthroplasty to damage-associated molecular pattern release and inflammasome activation.

The release of the DAMPs will directly target pattern recognition receptors (PRRs), mainly the Toll-like receptors (TLRs) and nucleotide oligomerization domain (NOD)-like receptors (NLRs) subfamilies [61]. Caspase-1, a protease, together with NLRs, participates in the creation of the inflammasome, which, once formed, along with TLR-based gene transcription induction, will stimulate the macrophages to start the production of IL-1β, a key cytokine for pro-inflammatory cascades [62] (Figure 1).

#### 3.1.2. Biomarkers

In a prospective study performed on 208 patients who underwent a total arthroplasty, it was found that high serum levels of HMGB1 were produced, which were directly correlated with an increase in C-reactive protein (CRP) and an increase in interleukin (IL-1β, IL-6), 1 day and 3 days after surgery, respectively, alongside a poor prognosis [63]. In another observational study, with 287 elderly patients, the same results as the previous mention were documented, but also correlated with another key element, anesthesia time [64]. It was shown that increased surgical time under general anesthesia can directly impact neural activity by increased HMGB1 serum levels, which promotes the production of CRP, IL-1β, IL-6, and TNF-α [64]. In some studies, cf-DNA is quantified from synovial fluid, both as a marker of local inflammation and, in chronic periprosthetic knee infection, as a diagnostic biomarker [65]. It is also a marker indicating the number of neutrophils implicated at the site of the traumatic event, depicting the density of neutrophil extracellular traps that appear, which, on their end, are correlated with an increased risk of infection [66], which makes cf-DNA a marker of interest for monitoring, although no intervention targeting it has yet been shown to reduce infection rates. Among DAMP-associated markers, IL-1β is of particular interest, since inflammasome activation by several DAMPs converges on its maturation and release [67]. Data on other biomarkers in the management of inflammation in total primary arthroplasties is scarce.

#### 3.1.3. Modulating the DAMP Response

The impact on inflammation in this specific pathway is determined by either the control over the levels of inflammatory markers produced by the macrophages after being indirectly stimulated by increased levels of DAMPs, or by managing the DAMP levels to halt macrophage activation through this mechanism.

##### Approaches Supported by Clinical Evidence

The following approaches have been evaluated in patients undergoing arthroplasty or comparable surgical procedures.

In a randomized controlled trial (RCT) performed on 60 patients, it was shown that repetitive transcranial magnetic stimulation (rTMS) can reduce the neural inflammation after total joint arthroplasty by directly reducing the postoperative levels of interleukins, TNF-α, and HMGB1 [68]. However, rTMS carries risks, as it can induce seizures or hyperacusis, which should be considered, especially if its intended use is only for anti-inflammatory purposes [68]. In another RCT performed on 119 elderly patients who underwent hip arthroplasty, it was shown that the administration of dexmedetomidine decreases the levels of all aforementioned inflammatory markers when combined with post-fascia iliaca compartment block [69]. While the main indication of dexmedetomidine is sedation and analgesic effects, it was proven to reduce inflammation; however, additional attention should be given to its prolonged administration for anti-inflammatory purposes, considering that cases of cardiac arrest were reported [70].

Evaluation of cf-DNA levels showed that a decrease in lesion area or radius or traumatic events caused a significant reduction in inflammation in patients undergoing various surgeries or interventions. In patients undergoing urological procedures, less extensive surgery was associated with a decrease in cf-DNA [71], suggesting that the magnitude of surgical trauma is itself a determinant of DAMP release. Similar to other increases in DAMPs, the tourniquet-caused ischemia also increases the level of cf-DNA, and a solution to manage this problem is the combined administration of antioxidant medication such as N-acetylcysteine and Deferiprone [72]. In essence, the use of N-acetylcysteine has been proposed for ischemia-induced inflammation, the only issue being that its pharmacological effect reaches a peak in about 1–2 h, which should be correlated with the tourniquet time in order to find a balance between ischemia-induced inflammation and the anti-inflammatory effect of the medication [73].

##### Preclinical and Experimental Approaches

The strategies described below target DAMP signaling directly but have so far been examined only in cell culture or in animal models.

Alongside rTMS, which is a method to directly reduce HMGB1 levels in human patients, data on auxiliary methods to directly target this DAMP are scarce in humans undergoing orthopedic interventions or surgeries. However, some existing substances were tested in vitro or in vivo on animal models that can target HMGB1. Glycyrrhizin is a natural product found in Licorice that has been widely used for viral diseases, such as hepatitis and coronavirus infections [74,75]. It was also shown that glycyrrhizin has an impactful anti-inflammatory activity mainly due to inhibiting HMGB1 [76]. In an in vitro study, the effects of glycyrrhizin were studied on chondrocytes from 40 patients with osteoarthritis [77]. This bioactive compound suppressed the activity of HMGB1, thus reducing the levels of IL-1β, while also suppressing the IL-1β-induced upregulation of HMGB1 [77]. In another study, in mouse in vivo models, glycyrrhizin could mitigate inflammation-caused osteolysis while promoting anti-inflammatory effects and anti-aging [78]. Given these important results, clinical studies using this licorice-derived compound on patients who underwent total joint arthroplasties should be further performed. Anti-HMGB1 antibodies reduced the expression of TNF-α in a murine periodontitis model [79]; whether this approach is applicable to periprosthetic inflammation has not been examined.

Deoxyribonucleases degrade cf-DNA directly and have been proposed as a means of reducing its inflammatory effects [80]. Studies on the use of DNase on human patients are scarce, but this therapy is used frequently as an anti-inflammatory in animal models. Although a physiologically active endothelial DNase exists [81], increasing its serum levels by external administration has proven effective in rats to reduce the inflammation, but more importantly, to reduce the levels of neutrophil traps [82,83]. Innovative solutions to scavenge and neutralize the cfDNA were studied. A team of researchers developed a drug-free nanomedicine by oxidizing polyethylenimine to diselenide mesoporous nanoparticles based on organic silica, with enhanced affinity to cfDNA, blocking its inflammatory pathways [84]. This nanomedicine was designed to be administered systemically for the treatment of intestinal inflammatory diseases. Local delivery to the periprosthetic space is proposed here as a hypothesis and has not been tested experimentally.

The status of the patients, other associated pathologies, risks, or ongoing treatment should always be considered when determining anti-inflammatory management, especially if the proposed solution could elevate other patient-related risks. Therapeutic approaches, adaptations, and clinical translations of these applications should be addressed in future research.

With the exception of repetitive transcranial magnetic stimulation and antioxidant administration, none of the strategies directed at DAMP signaling have been evaluated in patients undergoing arthroplasty. They are presented here as mechanistic rationale rather than as therapeutic options.

### 3.2. Acute-Phase Response Pathways

#### 3.2.1. Biomarkers and Clinical Correlations

Most notable in this direction are the C-reactive protein (CRP) and the erythrocyte sedimentation rate (ESR), which are frequently used as indicators of postoperative inflammation and the risk of associated periprosthetic joint infections [48,85]. Clinical data show that the increase in these two markers directly correlates with the immediate postoperative time, reaching a baseline after days or weeks. Given this predictable trajectory of acute-phase reactant markers, the management of inflammation can be done in a time-dependent manner [48,86]. Beyond the utility of CRP and ESR, another example that is frequently implicated in the inflammatory-response amplification of acute-phase markers is IL-6 [87]. Although this one is not routinely tested, given that it is most frequently present in viral inflammation, it can be an orientative marker for arthroplasties and postoperative management [85]. More importantly, to direct an inflammatory management in bone and joint-related disease, one needs to dose the markers obtained from the synovial fluid. Recent evidence highlights the use of an indicator-CRP to hemoglobin ratio (CHR) that may help delineate if an inflammation is septic or aseptic in nature [85].

Table 1 summarizes the reported postoperative behavior of these markers, together with the deviations from it that warrant further evaluation.

#### 3.2.2. Modulating the Acute-Phase Response

Alongside the aforementioned non-drug-based approaches, a direct way to manage these markers is by corticosteroid or NSAID medication, which is already a widely used and standard procedure [91,92]. Moreover, the systemic effects and anti-inflammatory potential of combined sedative medication should be explored, such as dexmedetomidine with propofol or opioids, which can be frequently found in the same therapies [93]. Safer pharmacological treatments to target the reduction in CRP, IL-1β, IL-6, and TNF-α exist, such as parecoxib, with antipyretic, analgesic, and anti-inflammatory effects that importantly reduce the recovery time after joint arthroplasties, especially if combined with recovery therapy under nurse guidance [94]. Tranexamic acid (TXA) has also become integral to perioperative care in primary arthroplasty, not only for its antifibrinolytic properties but also for its role in modulating postoperative inflammation [95]. Clinical studies in total hip and knee arthroplasty show that perioperative TXA reduces postoperative levels of inflammatory biomarkers, such as IL-6 and CRP, supporting its systemic anti-inflammatory effects [88,96,97,98]. Large meta-analyses and population-based studies confirm that TXA does not increase the risk of postoperative infection or thromboembolic complications, reinforcing its safety in primary arthroplasty [99].

## 4. Cytokine and Chemokine Signaling Pathways

### 4.1. Mechanistic Overview

One of the major pathways of inflammation is the system based on cytokines and their signaling mechanisms. Cytokines are proteins responsible for managing immune responses, inflammation, and stimulating cell growth and differentiation [40]. Chemokines, on the other hand, are a sub-specialized group of cytokines that specialize in regulating the communication between the inflammation-affected area and the immune cells, as reviewed in the context of cytokine and chemokine signaling [100]. Subfamilies of cytokines—interferons (IFN), interleukins, and TNFs, along with their messengers (chemokines) and other various growth factors and key regulators—ensure homeostasis by playing a key role in inflammation and immune response [100,101,102,103]. Cytokine signaling starts with their attachment to diverse cell surface receptors, leading to the activation of intracellular signaling cascades. Cytokine receptors activate Janus kinases (JAKs), which phosphorylate STATs and modulate gene transcription related to inflammation [104], bone remodeling, and immune reactions [105].

The second mechanism of cytokine signaling is the Nuclear Factor kappa-B (NF-κB) pathway, activated by pro-inflammatory cytokines such as IL-1 and TNF-α, which are quickly released on tissue injury [106]. There are two major NF-κB signaling pathways, canonical and noncanonical. The canonical pathway relies on the IKK complex-mediated breakdown of IκBα. The IKK complex comprises three subunits: two catalytic subunits, IKKα and IKKβ, and one regulatory subunit, NEMO (also known as IKKγ). The phosphorylation of the IκBα permits NF-κB dimers to start gene transcription [106]. The noncanonical NF-κB pathway is activated by specific ligands of the TNF receptor superfamily, such as CD40 and RANK. This pathway uses p100, which has its C-terminal IκB-like domain removed, thus producing the activated p52 subunit, which creates p52/RelB dimers. This alternative pathway acts like a highly specialized complementary signaling axis [106].

**Figure 2:**
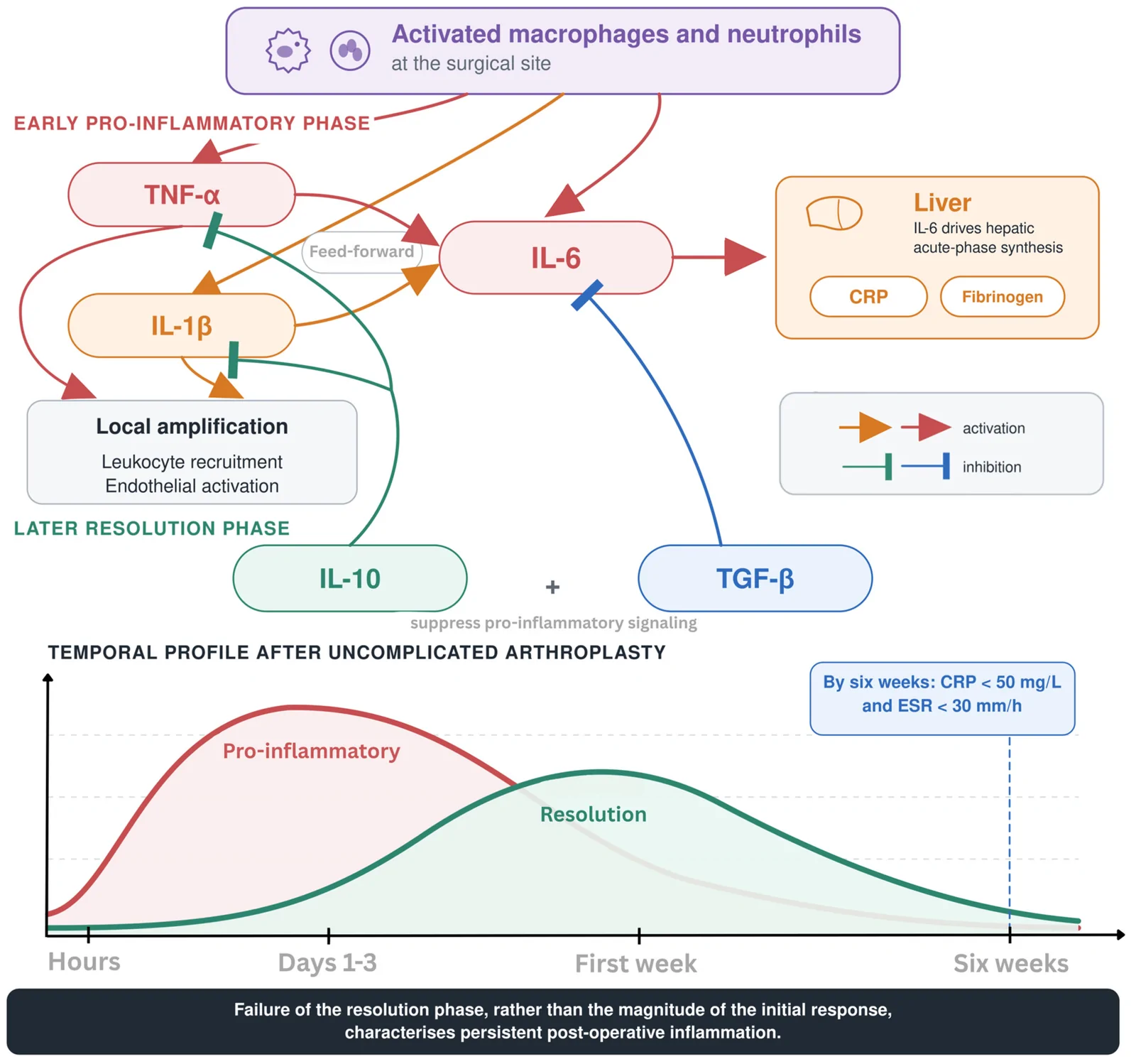
Cytokine signaling after primary total joint arthroplasty. TNF-α and IL-1β act locally and induce IL-6, which drives the hepatic acute-phase response. IL-10 and TGF-β suppress the pro-inflammatory arm during the later resolution phase. The lower pane shows the temporal profile after uncomplicated surgery, with CRP falling below 50 mg/L and ESR below 30 mm/h by six weeks.

The third mechanism of cytokine signaling is the mitogen-activated protein kinase (MAPK) [107] pathway, which modulates cell proliferation, growth, apoptosis, and cytokine production; therefore, it is important in the onset of inflammation [108]. The MAPK cascade converts extracellular stimuli, such as wear particles or cytokines, into specific cellular responses. It is a three-tier kinase relay; the first kinase in the MAPK signaling cascade is a mitogen-activated protein kinase kinase kinase (MAPKKK) that initiates the phosphorylation of a MAPKK, which in turn activates MAPK (Extracellular Signal-Regulated Kinase (ERK), c-Jun N-terminal Kinase (JNK), p38). Once activated, after nuclear translocation, MAPKs also initiate inflammation via phosphorylation of the transcription factors [109]. Wear particles act as one such extracellular stimulus; their role in periprosthetic osteolysis is considered in Section 5.3. [110]. The interaction between these mediators, and the temporal profile that results from it, is summarized in Figure 2.

### 4.2. Targeting Cytokine Signaling

None of the strategies described in this section has been evaluated in patients undergoing arthroplasty. They are presented as mechanistic rationale derived from cell culture and animal models.

Managing the inflammation on the canonical pathway of the NF-κB-based mechanism was tested in vivo on animal models by using NEMO-binding peptide (NBD). It was shown that NBD can significantly reduce synovial inflammation as well as bone and cartilaginous degeneration while inhibiting osteoclastogenesis and promoting osteointegration; these findings derive from models of rheumatoid synovitis, osteomyelitis, disk degeneration, and titanium implant integration rather than from periprosthetic osteolysis [111,112,113,114,115].

For the noncanonical pathway, a small inhibitor of p100-to p52-based inflammatory response was tested and proven successful in reducing oxidative stress and inflammation in the liver. Although not yet tested in the field of orthopedics, this small inhibitor, named B022, could be adapted to promote its activity by directly targeting this specific mechanism of NF accumulation [116]. Overexpression of the noncanonical pathway leads to increased levels of pro-inflammatory cytokines associated with bone resorption and aseptic loosening in post-arthroplastic surgeries [110]. These pathways are extensively studied for their possibilities in the development of anti-inflammatory drugs for diseases such as rheumatoid arthritis, aiming at NF-κB [106].

Erythromycin is a macrolide antibiotic that inhibits inflammatory osteoclastogenesis in mice by modulating the NF-κB pathway. It decreased tissue inflammation induced by UHMWPE by reducing IL-1β and TNF-α expression, and diminished gene transcription of CPK, RANK, and RANKL, and also prevented bone collagen loss. Erythromycin administration for the management of inflammation in humans after arthroplasty has not been investigated [110,117]. Tetracycline had similar effects as erythromycin in mice, inhibiting inflammatory osteolysis by a significant decrease in RANK, RANKL, and a minor decrease in TNF-α, as well as MMP-9 in Ti wear-debris-induced inflammation. Its use in patient care practice, post-total joint replacement surgery, is yet untested, but may reduce the risks of complications [118]. Melatonin, or 5-methoxy-N-acetyltryptamine, is a hormone secreted by the pineal gland [119] that can preserve the structural integrity of β-catenin, with levels significantly reduced upon exposure to Ti particles both in vivo and in vitro, thereby contributing to increased periprosthetic osteolysis. β-catenin inhibits the NF-κB signaling pathway and reduces IL-1β, IL-6, and TNF-α. On the other hand, melatonin modulates levels of osteoprotegerin (OPG) via activation of the Wnt/β-catenin signaling pathway. Although melatonin appears to be a promising therapeutic drug for post-arthroplasty inflammation management, its clinical application may be limited by perioperative antibiotic administration, which blocks its effects [120,121].

In conclusion, cytokines play a role in the wear-debris-induced inflammation following arthroplasty, modulating osteoclast activation and bone resorption. Pathway-targeted modulation of NF-κB signaling, rather than global suppression of the inflammation, is a concept which could be useful for limiting wear-debris-induced bone resorption. Whether it can reduce aseptic loosening or improve implant survival in patients remains to be established.

## 5. Bone Remodeling-Based Pro-Inflammatory Pathway

### 5.1. Mechanistic Overview

#### 5.1.1. The RANK/RANKL/OPG Axis

Through the physiological process of bone remodeling, bone changes shape, size, or density to adapt its properties to mechanical and chemical stress [122]. Both remodeling and post-traumatic healing involve a primary stage of inflammation that recruits cells via acute-phase cytokines such as IL-1, IL-6, and TNF [123]. However, exaggerated or systemic inflammation significantly impacts these properties. Arthroplasty success rates depend on many factors, but more significantly on the integration of the prosthesis in the host bone. After insertion, the bone must be able to mold itself around and inside the prosthesis, a process called osteogenesis. Faulty osteogenesis translates to a higher risk of implant failure, such as loosening or fracture [124]. In terms of inflammation pathways, the RANK/RANKL/OPG pathway is responsible for osteoclast activation and activity by inducing the production of osteolytic enzymes [125]. With RANKL being an inducer and OPG an inhibitor of this pathway, options for managing the immune response, osteoclast activation, and osteolysis could arise from interacting directly or indirectly with these molecules.

The RANK/RANKL/OPG pathway is best known for its influence over bone resorption as a result of inflammation [126]. RANK and RANKL are expressed on the surface of osteoblasts and osteoclasts, respectively. In inflammation, the expression of RANKL is increased due to interaction with cytokines, glucocorticoids, PTH, and other substances. RANKL then binds to RANK on osteoclasts, stimulating the production of their matrix-degrading enzymes. OPG inhibits this pathway by acting as a decoy for RANKL.

#### 5.1.2. Macrophage Polarization

Macrophages at the bone–implant interface display a dynamic phenotypic spectrum between the pro-inflammatory M1 and reparative M2 states, and their immune surveillance response to wear debris fails when the transition to an M2 phenotype does not occur; the persistence of these microparticles locks the cells in the chronic M1 state, triggering the periprosthetic osteolytic cascade [127,128]. An initial M1 response participates in debris clearance and early wound healing, but the subsequent bone generation is dependent on the shift towards an M2 phenotype [129]. Preclinical data show a time-limited rather than prolonged shift: M1 activity prolonged beyond a critical time window proved detrimental to osseointegration in an experimental titanium implant model (the optimal time window for a conversion to M2 was between days 3 and 7) [130].

In primary human macrophages, particles of PMMA and hydroxyapatite polarized the cell towards M1 in a Syk- and MAPK-dependent manner, the activation of which was directly targeted by a Syk inhibitor or MAPK inhibitor [131]. Thus, the activity on MAPK seemed to be central (as highlighted in Section 4 of this paper). Inflammatory response to wear debris is not simply one toward M; specifically, cobalt–chromium–molybdenum debris forces macrophage polarization onto a distinct phenotype carrying a mixed M1/M2 transcriptional signature, which forces the macrophages to be chronically inflamed and hypersecretory [132].

**Figure 3:**
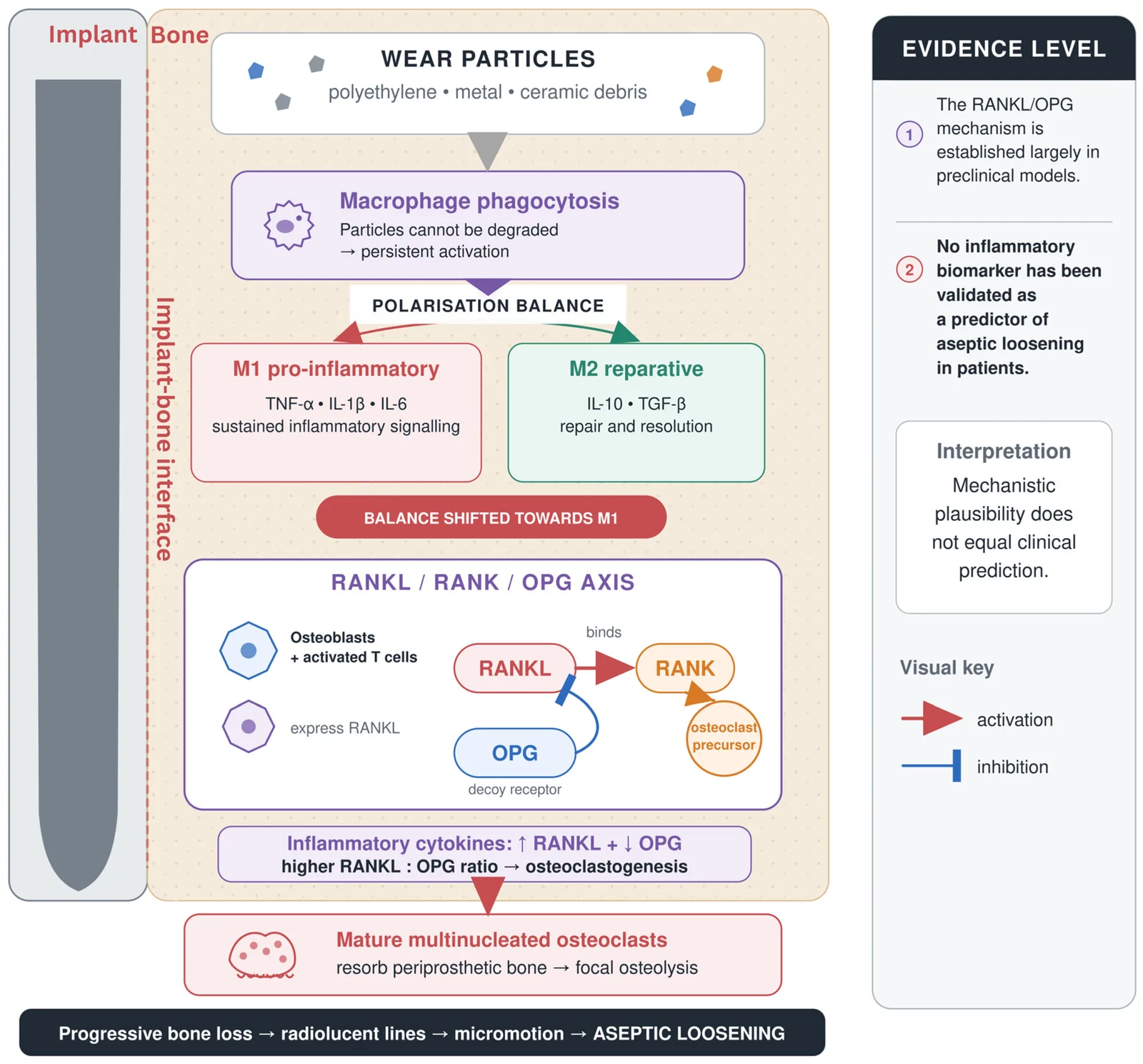
Osteoimmunological mechanism of periprosthetic osteolysis. Wear particles are phagocytosed but not degraded, sustaining macrophage activation and shifting polarization towards the M1 phenotype. Inflammatory cytokines raise RANKL and lower osteoprotegerin, and the resulting ratio determines osteoclast differentiation and focal bone resorption. The side panel states the level of evidence supporting this mechanism.

The downstream result converges on the axis described in Section 5.1.1. Titanium particles were responsible for the induction of M1 polarization via p38α/β and generated an OPG/RANKL imbalance favoring osteoclastogenesis [133]. This appears modifiable: in an in vivo polyethylene-particle murine model, IL-4 administered after osteolysis had already been established reduced M1 and osteoclast numbers, increased M2 and osteoblast numbers, and restored lost bone [134], indicating that failed polarization is a causal and potentially reversible contributor. The sequence from wear particle regeneration to aseptic loosening and the level of evidence supporting it is presented in Figure 3.

#### 5.1.3. Adaptive Immune Cells and Bone

RANKL is not produced exclusively by cells of the osteoblast lineage. Osteocytes, stromal cells, macrophages, neutrophils, and both T and B lymphocytes can express it, and this expression is amplified by TNF-α, IL-1β, IL-6, and IL-17A (in parodontitis animal models) [135], which places adaptive immunity within the same axis described in Section 5.1.1. In the arthroplasty setting, direct evidence includes the spontaneous osteoclastogenesis observed in peripheral blood mononuclear cells of patients with established periprosthetic osteolysis when cultured in vitro [136]. Osteoclastogenesis was eliminated by both RANK-Fc and T-cell depletion, indicating it was a T-cell-dependent process that was mediated via RANKL [136]. The presence of both T cells and cells with a CD8+ CD25+_FoxP3+ regulatory cell phenotype alongside osteoclasts within periprosthetic tissue suggests a role in early activation as well as later suppression [136]. Th17 response has been observed in a patient with a ceramic-on-ceramic total hip arthroplasty, where neck-rim impingement induced an inflammatory IL-17-responsive CD4+ infiltrate [137]. However, this is a result of a single patient, and should not be interpreted in a general way.

Beyond these observations, arthroplasty-specific data remain limited. The reciprocal regulation of osteoclastogenesis by Th17 and regulatory T cells, and the role of B lymphocytes as a source of both RANKL and osteoprotegerin, are well characterized in rheumatoid arthritis, periodontitis and postmenopausal bone loss, but have not been systematically examined at the bone–implant interface.

#### 5.1.4. Osteoblast–Osteoclast Coupling

For proper bone remodeling to occur, the physical and biological link between bone formation (replacing lost bone) and destruction of bone by osteoclasts must be maintained: there must be equilibrium in where and when the two occur, which can be broken by the progression of osteoporosis [138]. Coupling operates in part through direct contact, whereas ephrinB2 expressed on osteoclasts engages EphB4 on osteoblasts, and this bidirectional signaling suppresses resorption while promoting formation, whereas ephrinA2/EphA2 acts in the opposite direction [139,140]. There are also contributions from soluble mediators, in particular, sphingosine-1-phospate which stimulates proliferation of osteoblasts and has been demonstrated to control osteoclast precursor recruitment to resorption sites, although this coupling has been characterized in bone remodeling generally rather than at the periprosthetic interface [141]. Recent findings suggest that pre-osteoclasts can themselves be the mediator of coupling via their secretome, including sphingosine-1-phosphate, PDGF-BB, and afamin [142]. In addition, it suggests an anabolic action of pre-osteoclasts is dissociable from that of resorption [142], an observation relevant to the rebound phenomenon described for Denosumab in Section 5.4.

The pro-inflammatory cytokines inhibit the expression of Runx2 and osterix; they enhance the Wnt inhibition via sclerostin and DKK1, and modulate the signaling of ephrinB2/EphB4 and semaphorin, so resorption happens while the compensatory formation phase fails [135]. This mechanism has not been characterized directly in the periprosthetic environment, and is derived from other models of inflammatory bone loss.

### 5.2. Biomarkers

An animal study has shown that increased pro-inflammatory activity lowers the quality of bone healing [143]. Conversely, fracture healing was accelerated in mice lacking an adaptive immune system, indicating that lymphocyte-driven inflammation can itself delay repair [144]. The ratio of RANKL to OPG is a useful metric in evaluating the state of activity in osteoclasts. Managing the activity of osteoclasts is crucial in the management of inflammation secondary to arthroplasty, since osteolysis affects implant viability [127]. When discussing bone inflammation, the amount is proportional to the damage.

### 5.3. Clinical Correlates: Aseptic Loosening, Periprosthetic Osteolysis and Implant Survival

Primary total joint arthroplasty is a long-lasting procedure. Pooled data from eight registries covering 1,904,237 primary total hip arthroplasties report survivorship of 93.6% at 20 years [145]. The failures that do occur are already discussed mechanistically in previous sections. In another series of 7941 primary total knee arthroplasties, 56.6% of the revisions were accompanied by synovitis, osteolysis, and component loosening, due to polyethylene wear, 5 years after the procedure [146]. This corresponds to the macrophage and RANKL-driven cascade described above, observed at revision surgery rather than in an experimental model. The survivorship reported in that series was lower than expected, and the implant was subsequently recalled.

Periprosthetic bone loss at the implant–bone interface allows micromotion, which will eventually lead to early migration. To assess this, a group of researchers used radiostereometric analysis (RSA) at two years follow-up to predict aseptic loosening in 339 patients, and a lower ten-year revision was reported in the RSA-tested group (4.4% compared to 5.5% in the control group) [147]. In general, this process is detected radiographically as progressive radiolucent lines and focal osteolysis, although such findings are not themselves equivalent to failure: in a cohort of 585 primary knees followed for 11.2 years, radiolucent lines were present in 5.8% (after a mean of 2.4 years) without measurable migration, while aseptic loosening occurred in only 0.8% [148].

Whether this pathway is susceptible to pharmacological modifications remains unresolved. A recent meta-analysis found that the use of perioperative bisphosphonate (RR 0.67, 95% CI 0.54–0.83) reduced all-cause revisions, but showed no effect on aseptic loosening or osteolysis [149]. To conclude, none of the reviewed biomarkers in this paper have been validated as a predictor of aseptic loosening in a clinical cohort.

### 5.4. Pharmacological Modulation of Bone Remodeling

Since inflammation is a necessary factor in any bone-remodeling process, we can infer that lowering the physiological inflammatory response with NSAIDs should negatively affect bone healing [150]. A baseline that we can refer to is a bone fracture in an otherwise healthy patient. In arthroplasty, inflammation levels are higher than the baseline due to factors such as tissue damage from the surgery, ischemic damage from tourniquet use, and thermal damage from cement setting. Furthermore, chronic foreign-body inflammation is inevitable in arthroplasty patients due to prosthetic wear and phagocytosis of liberated metal particles. To ensure the level of inflammation that promotes healing and osteogenesis, while also maintaining it below the point at which inflammation becomes harmful, a very precise intervention in the RANK/RANKL/OPG pathway is necessary. The overall goal would be to skew the RANKL to OPG ratio in favor of OPG [32,151]. To achieve this, a few therapeutic approaches can be employed. None of the agents discussed below is licensed for the prevention of periprosthetic bone loss; the evidence derives from their use in osteoporosis and from studies of periprosthetic bone mineral density. The most studied one involves Denosumab, a monoclonal antibody that binds to RANKL, effectively assuming the decoy role that OPG physiologically accomplishes [152]. This alters the osteolysis/osteogenesis balance in favor of the latter, preserving periprosthetic bone stock by maintaining bone mineral density (BMD) (at a 0.7% loss of BMD against a control group’s 20%) [153]. Since this pathway is sensitive to parathyroid hormone, researchers have attempted to use an anabolic therapy. Initially used as a treatment for osteoporosis, PTH analogs such as intermittently administered PTH 1-34 have shown promising effects in mitigating osteoclast hyperactivity by increasing the expression of OPG and increasing Wnt/b-catenin signaling [154]. Its dual effect has great potential, although caution is needed as high or frequent doses of PTH analogs could stimulate osteoclasts [154].

A third approach consists of administering strontium ranelate, a drug formerly used for osteoporosis. This now-discontinued drug acts on both branches of the RANK/RANKL pathway activation. It stimulates osteoblast replication and OPG expression while also reducing RANKL expression and promoting osteoclast apoptosis [154]. This approach carries risks, as the main reason behind its discontinuation lies in its cardiovascular risks in patients with a cardiac history [155]. These agents act on osteoclast activity through the RANK/RANKL/OPG axis, but none has been shown to reduce aseptic loosening in patients. Denosumab stops being helpful when treating chronic patients because of the accumulation of RANKL and because of rebound. Patients treated with Denosumab showed a threefold increase in circulating RANKL levels 3 months postoperatively, which persisted for another 3 to 9 months [156]. If Denosumab treatment is interrupted, circulating RANKL will stimulate osteoclasts intensely, leading to abrupt BMD loss [156].

All the aforementioned substances pose specific risks when considered in the surgical context. Denosumab, for example, has no known drug–drug interaction with CYP3A4-metabolized drugs (midazolam, alfentanil, fentanyl) [157]. However, as an osteoclast inhibitor, it carries the risk of hypocalcemia, which could pose significant operatory risks in patients with low calcium or vitamin D deficiency (neuromuscular blockade, cardiac conduction). Strontium ranelate has known and documented cardiovascular adverse effects, such as thromboembolism or myocardial infarction. PTH analogs also alter blood calcium balance, so an anesthesiologist should consider this when sedating a patient.

Managing acute and chronic inflammation in patients undergoing arthroplasty involves a fine balance between allowing physiological processes to take place while keeping them from being exaggerated. Physicians tackling this problem have a documented and proven leverage on these processes using various therapies that skew the balance of osteoblast/osteoclast activity.

## 6. Infection-Driven Inflammatory Pathways

**Figure 4:**
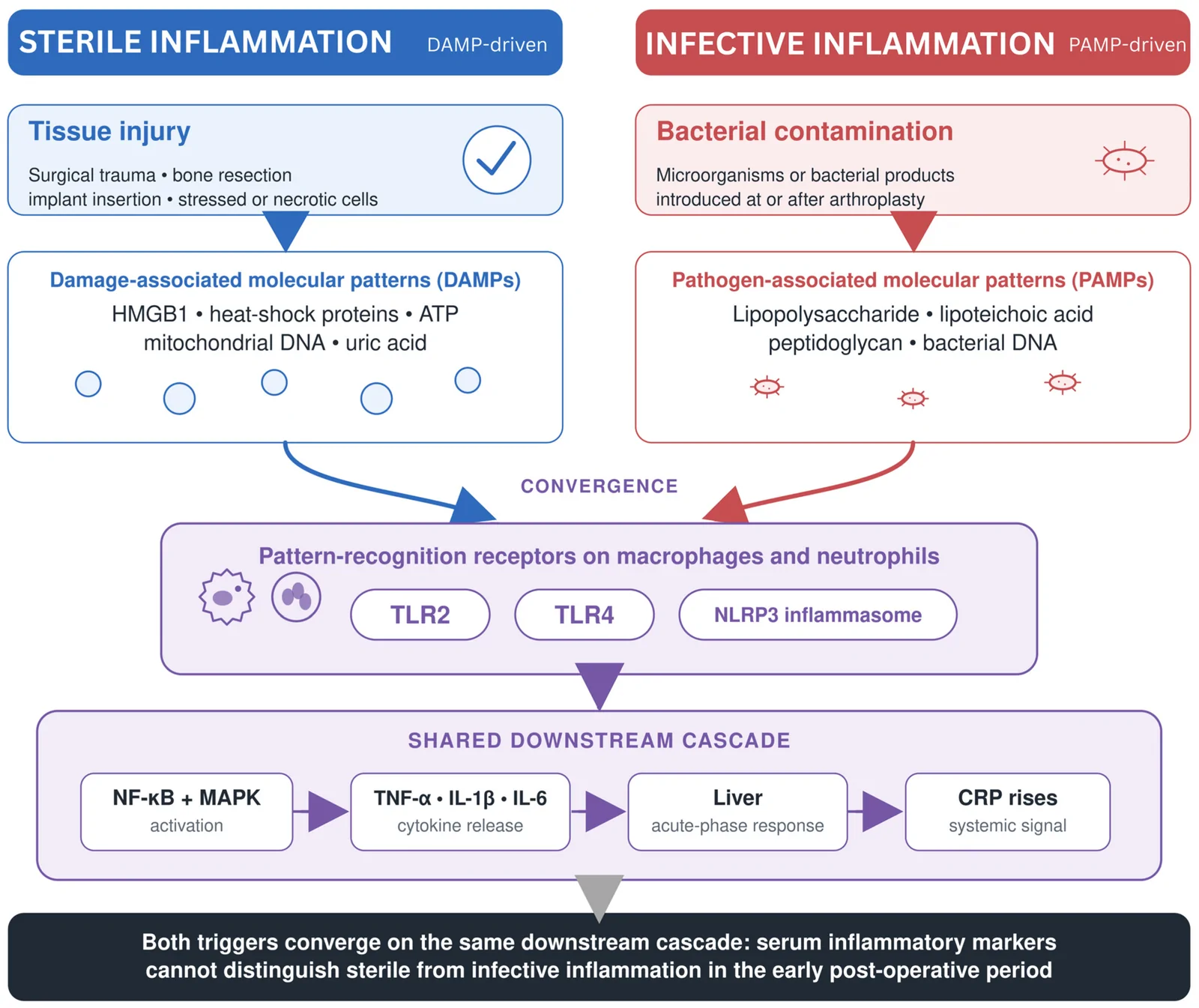
Sterile and infective inflammation after primary total joint arthroplasty. DAMPs released by surgical trauma and PAMPs introduced by bacterial contamination are detected by the same pattern recognition receptors and converge on a shared downstream cascade, which is why serum inflammatory markers cannot distinguish the two in the early postoperative period.

Infection-driven pathways are the body’s physiological response to microbes [158]. They work based on encoded receptors that recognize surface molecules on microorganisms (like flagellin, lipopolysaccharides, peptidoglycan, profilin, or zymosan) or segments of DNA/RNA [159] called pathogen-associated molecular patterns (PAMPs). These pattern recognition receptors (PRRs) are present on innate immunity cells. Their activation starts the PAMP pathway, NF-κB, which engages the same NF-κB, MAPK, and JAK-STAT cascades described in Section 4, and the same inflammasome-dependent maturation of IL-1β described in Section 3.1.1, raising circulating levels of IL-1β, Il-6 and TNF-α and recruiting neutrophils and monocytes [160,161,162]. The exaggerated form of this amplification is called a cytokine storm. Foreign DNA and RNA are detected via cytosolic nucleic acid sensors (cGAS-STING): a cyclic GMP-AMP synthase (cGAS) and a cyclic GMP-AMP receptor stimulator of interferon genes (STING). The distinction between the two triggers, and the point at which they converge, is presented in Figure 4.

Understanding these pathways is crucial for effective management of infectious inflammation, an overlying condition that can alter the regenerative processes occurring after arthroplasty.

One of the primary mechanisms by which the immune system detects pathogens is by recognizing atypical structural conformations through pattern recognition receptors, such as Toll-like receptors (TLR), NOD-like Receptors (NLR), C-type Lectin Receptors (CLR), and RIG-I receptor [163]. Addressing the inflammation alone and failing to detect the underlying microorganisms may facilitate their proliferation. Taking this into consideration, a method of controlling the inflammation is by reducing its chances of developing. Antibiotic prophylaxis greatly reduces pathogen acquisition during arthroplasty, as shown in a nationwide study of elbow arthroplasty [164]. However, compared with native joint infections, PJIs generally present with a lower microbial burden, which may make pathogen detection more challenging [165]. Moreover, once bacteria infect a joint, the biofilm makes it inaccessible to therapies [166,167]. Most perioperative infections can be categorized based on their time of appearance and type: those that appear within four weeks of surgery are generally caused by *Staphylococcus aureus*, β-hemolytic streptococci, and *Enterococcus* spp. Between 3 and 12 months post-op, coagulase-negative staphylococci, *Cutibacterium acnes*, and enterococci are most likely to cause infections. One to 2 years after surgery, hematogenous infections are most likely to occur. These involve pathogens like *Staphylococcus aureus*, coagulase-negative staphylococci, enterococci, and occasionally Gram-negative bacilli [168]. It should be noted that the route of drug administration (systemic or local) plays an important role. Meta-analyses show that local administration has lower periprosthetic joint infection (PJI) rates than IV administration [169,170,171]. The empirical antimicrobial therapy is focused on staphylococci and Gram-negative bacilli, being the most commonly isolated pathogens [172]. The most frequently used antibiotics are cefazolin [173] and clindamycin, with cefazolin having higher efficacy [174].

Prevention measures are meant to reduce the risk of postoperative infection, and this goal can be achieved by tackling the sources of infection and preventing new ones. Screening and decolonization for *S. aureus*, for example, is advised, as meta-analyses show that it reduces surgical site infection in orthopedic surgery, including elective arthroplasty [175]. As with any surgery, preventive protocols are mandatory and are a proven way of reducing the spread of perioperative infections. Clinically, PJIs manifest inconsistently, with joint pain as the common denominator between cases [176]. Local signs of inflammation, such as swelling or erythema, may be present, while systemic signs, like fever, are frequently absent [177,178]. Pathognomonic signs also exist, such as a draining sinus duct; however, its sensitivity is low [178].

Infectious pathogens should be treated separately from non-infectious inflammation. As mentioned above, inflammation is beneficial within boundaries, which are exceeded when infection is also contributing to inflammation. Antibiotic therapy should be carefully selected and administered if an infection is detected via culture and antibiogram. Differential diagnosis with non-infectious inflammation should be a priority. Laboratory findings, cultures, and imaging are to be referred to, while keeping in mind that a lower threshold is more indicative of PJI than other osteoarticular inflammatory diseases [179]. Imaging has limited diagnostic application, but can show erosions, abscesses, or prosthetic loosening.

## 7. Discussion

### 7.1. Biomarker Performance in Practice

Systemic inflammatory biomarkers typically measured in serum or plasma include CRP, ESR, IL-6, fibrinogen, D-dimer, and procalcitonin. These inexpensive tests are widely available, but each has limitations. CRP and ESR quickly rise after tissue injury but lack specificity in the early postoperative period and can be influenced by other inflammatory conditions [180]. IL-6 peaks earlier and may improve sensitivity, but its assays are more costly and less accessible [181]. Fibrinogen may offer diagnostic performance similar to CRP at a lower cost [182]. D-dimer and procalcitonin have poor specificity and are affected by surgery or thrombotic status [183,184]. In contrast, synovial fluid markers such as white blood cell (WBC) count, polymorphonuclear neutrophil (PMN) percentage, synovial IL-6, alpha-defensin, leukocyte esterase, and calprotectin better reflect local periprosthetic inflammation and generally provide higher diagnostic accuracy [185]. However, joint aspiration is required to obtain synovial fluid, which may be limited by low fluid volume or patient discomfort. Some assays, such as alpha-defensin, are expensive, while others, like leukocyte esterase and calprotectin, are inexpensive and rapid [186,187]. Synovial IL-6 and calprotectin show sensitivities and specificities above 0.87 and 0.90, respectively, but are less widely available [181,187]. WBC count and PMN percentage require careful threshold selection to balance sensitivity and specificity, and results may be confounded by other inflammatory causes [185]. Table 2 presents key inflammatory biomarkers currently utilized in clinical practice, as well as additional emerging markers that may be considered for monitoring inflammation levels following arthroplasty.

Several of the biomarkers discussed here are not specific to infection. HMGB1, cell-free DNA, and IL-1β are released during sterile inflammation, in which tissue damage alone, without a pathogen, activates the same innate sensing pathways [210]. Neutrophil extracellular traps are likewise now recognized in a range of non-infectious conditions [211]. Degradation of the extracellular matrix generates fragments that are themselves biologically active and can sustain local inflammation [212,213]. The diagnostic value of these markers therefore lies in capturing overlapping injury and remodeling pathways rather than in identifying a specific disease entity [214]. When comparing serum and synovial markers, the results are less straightforward than they seem. The results are dependent on the clinical questions asked. Serum IL-6 outperforms CRP in acute periprosthetic joint infection, whereas CRP retains the advantage in chronic presentations [90]. Thus, they cannot be generally ranked over each other. The same logic applies to the purpose of the test: alpha-defensin is more effective for excluding infection rather than confirming it, which makes it more valuable in a screening role [197]. The efficiency of their use is conditional on timing and on whether the clinician is seeking to rule infection in or out.

Similar difficulties lie in cut-offs. Synovial cut-offs are generally not transplantable from one procedure to another; specifically, TKA needs a higher cell count of polymorphonuclear cells and leucocytes than unicompartimental knee arthroplasty [193,194]. The published definitions themselves disagree, since the EBJIS 2021, ICM 2018 and IDSA 2013 criteria differ both in their thresholds and in the weight assigned to individual tests, and classify the same patients differently [178]. A cut-off quoted without its definition, its joint, and its population is therefore of limited use. The largest gap is between the mechanism and its predicted outcome. Agents, such as bisphosphonates and Denosumab, that affect the RANK/RANKL/OPG axis result in the predicted periprosthetic bone outcome, but none of them can be demonstrated to prevent aseptic loosening in patients [149,156].

### 7.2. A Practical Framework for Biomarker Interpretation

**Figure 5:**
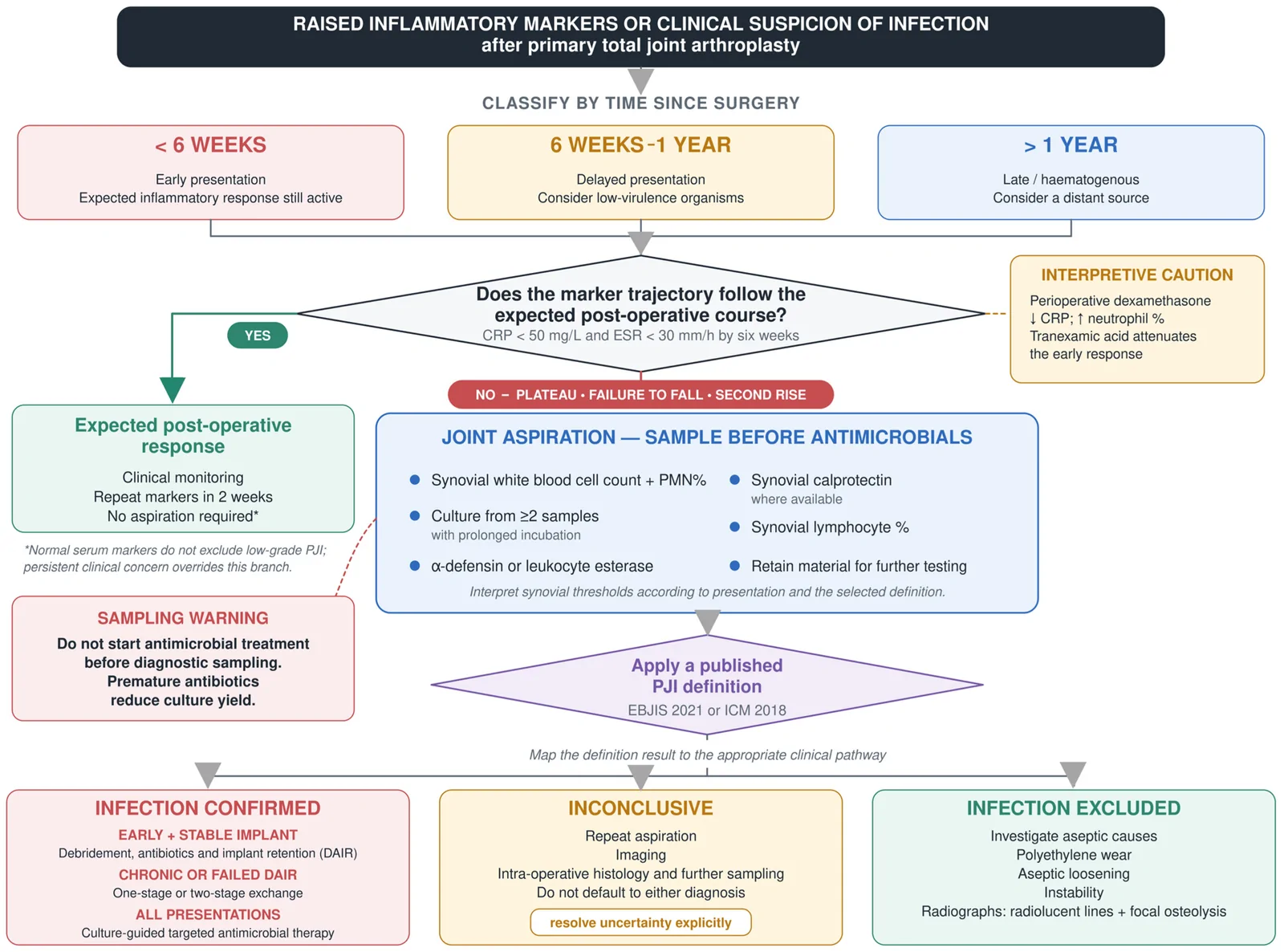
Clinical decision-making algorithm for suspected periprosthetic joint infection after primary total joint arthroplasty. Thresholds differ between the EBJIS 2021 and ICM 2018 definitions and derive largely from revision cohorts; they should be applied with caution in the first weeks after primary surgery.

The preceding sections describe the thresholds of various biomarkers and the mechanisms behind the rising values. However, what the clinician faces is a single patient with a raised marker and a decision to make. Figure 5 sets out a framework that links the kinetic reference points of Table 1 to the diagnostic thresholds of Table 2 and to subsequent management. The framework highlights two questions. The first is temporal, since infections presenting within six weeks, between six weeks and one year, and beyond one year differ in their likely organisms and in the treatment options that remain available. The second is whether the observed trajectory conforms to the expected postoperative course. All the results should be interpreted against a published definition rather than against isolated cut-offs, since the EBJIS 2021 and ICM 2018 definitions differ in both their thresholds and the weight given to individual tests [178].

### 7.3. Limitations of Current Evidence

There are several limitations present in this narrative review. Firstly, the mechanistic elements described in Section 4 and Section 5 (pattern recognition signaling, RANKL/OPG axis, coupling between osteoblasts and osteoclasts) are primarily based on data obtained from animals and from laboratory experiments, whereas the clinical biomarker data presented in Section 3 are drawn from patient populations and data collected in real-life settings. These two groups of evidence are not equivalent in strength, and the mechanistic pathways should be read as plausible explanations for the clinical observations.

Secondly, the thresholds reported at the various stages are almost exclusively reported in cases of suspicion of infections and revisions, rather than those undergoing initial arthroplasty. Moreover, where data are available for primary arthroplasties, they relate almost solely to total knee arthroplasties. Comparable data after hip arthroplasty are sparse. Thus, extending these thresholds to early post-primary surgery is an extrapolation.

Thirdly, no inflammatory marker has been validated as a predictor for aseptic loosening. Although it is mechanistic, it is well-established that particle-induced inflammation contributes to osteolysis around the prosthesis. However, this finding has not led to the identification of a marker that identifies patients at risk of this complication prior to radiographic evidence.

Fourthly, common perioperative interventions confound the interpretation of early postoperative markers and are inconsistently reported. Corticosteroids, tranexamic acid, tourniquet use, and thromboprophylaxis each modify the inflammatory response, yet very few of the studies cited here present these results accordingly.

Finally, this review is narrative in design. Studies were identified through the search described in Section 2, but no formal risk-of-bias assessment or quantitative synthesis was undertaken. Relevant work published elsewhere may not be represented, given that the search was done in the PubMed database.

### 7.4. Future Perspectives

In the future, total joint arthroplasty surgeries should aim for a minimally invasive approach in order to limit the amplification of the inflammatory response due to surgical workmanship [215,216,217]. At this point, physicians are required to manage postoperative inflammation (acute and chronic) using, in most cases, drug-based therapies. Within the current understanding, the management of inflammation does not mean targeting its absence, but rather allowing the organism to work toward healing itself while keeping it balanced, to reduce the risk of infections or other complications and pain. All the inflammatory pathways discussed above are not functioning independently; rather, it is a complex network that autoamplifies, concluding with an increased level of inflammatory markers, with systemic and local responses.

Furthermore, inflammation and implant viability are directly linked and vary inversely proportional. It is apparent that stimulating osteogenesis is more beneficial than inhibiting resorption; therefore, combined therapies like strontium ranelate or therapies that focus on osteoblasts are more productive. There are also complementary therapies that should be investigated in combination with a substance-focused one, such as Low-Intensity Pulsed Ultrasound (LIPUS), for which studies report promising results in terms of bone healing and regeneration [218], Pulsed Electromagnetic Field (PEMF) or mechanical vibration therapy, which improved healing, callus formation and mineralization in animal models [219].

Current approaches aim at the use of global suppression of inflammation through corticosteroids and NSAIDs [220,221], or optimized analgesic medication. While effective, the systemic response could bring less beneficial effects than the risk or the stress that is induced in the organism. Future strategies should aim to shift from these current approaches to pathway-targeted approaches, as exemplified in the sections of this paper. As an example, for DAMPs, it is possible to target HMGB1, ATP, histones, heat shock proteins, and markers that drastically increase after arthroplasties [47,163]. To bypass the risks of systemic administration, novel delivery vectors can be designed. Local, or periarticular, such as injections, coating, or hydrogel-based local drug delivery, could be some of the possibilities. Many non-specific biomarkers are increased in any kind of traumatic intervention, such as surgery. Other approaches to manage inflammation caused by usual markers (such as interleukins, chemokines, CRP, etc) may be represented by the analysis of which plays the most significant role and implementing a therapy directly targeting that major in-play marker.

Strategies of preoperative treatments with antioxidants, anti-inflammatories, or even antibiotics could be included in the standard management of patient preparation. Restricting tourniquet use to limit ischemia and reducing the elements that cause accidental cell death can be targets for surgical teams. Moreover, the development of biomarkers that could differentiate aseptic from pathogen-induced inflammation could be another future research direction, thereby classifying the inflammation with DAMP or PAMP starting points.

Pharmacological manipulation of bone remodeling should be measured against loosening or revision and not on periprosthetic bone mineral density, which has remained a parameter that has never been correlated to survival. In addition to new therapy agents, several methodological developments can be expected to change biomarker research in the field. Individual, cellular characterization of the periprosthetic membrane provided by single-cell RNA sequencing and spatial transcriptomics can differentiate the macrophage, fibroblast, and lymphocyte types that bulk tissue analyses average together, with similar technologies already used to study periprosthetic tissue for the comparison of infection against aseptic loosenings [222]. Profiling of immune cells combined with multi-omics integrative analysis could help identify the characteristic signs distinguishing aseptic from septic inflammatory reactions before even single-circulating biomarkers detect any change.

Machine learning models utilizing a mix of clinical, serologic, and synovial factors have been introduced for the diagnosis of periprosthetic joint infection; however, recent systematic review authors identify a lack of external validation in a majority of these algorithms [223]. Smart wearable devices may supplement regular blood sample taking in order to enhance postoperative surveillance systems; sensors measuring gaits (walking pace) have shown their applicability to distinguish well between overall functional results after primary knee replacement [224]. None of these technologies has so far managed to prove itself as applicable to the perioperative inflammation assessment in primary arthroplasty series; each, however, addresses a limitation of the biomarker panel currently available.

## 8. Conclusions

A better understanding of the inflammatory sources after arthroplasty, their biochemical pathways, and their effects on osteoimmunology can empower physicians with knowledge of why non-infectious inflammation is beneficial to a degree. A detailed explanation of these mechanisms helps recognize how they can be influenced and how these alterations could influence the evolution of bone lesions. While it is the surgeon’s or general practitioner’s choice that determines the outcome of an intervention, managing inflammation effectively can ensure that arthroplasty recipients do not suffer from complications such as stress fractures or aseptic necrosis. The sensitive demographic of arthroplasty implies the necessity to maintain the revision rates as low as possible. This paper provides a shared perspective on each inflammatory pathway involved in the postoperative period of total joint arthroplasty. What remains unresolved is the methodology to accurately identify which inflammatory pathway is predominant after a given orthopedic procedure. Future studies should assess methods to reduce anti-inflammatory treatments that act with less efficiency but on multiple pathways, in favor of identifying targeted treatments for a more optimal response.

## Figures and Tables

**Table 1:** Reported postoperative behavior of inflammatory markers relevant to the assessment of suspected periprosthetic joint infection. The source population differs between rows and is stated for each.

Marker	Reported Postoperative Values and Source Population	Reported Trajectory	Observation Relevant to Clinical Interpretation	Ref
CRP	Day 3: 33.32 ± 11.56 mg L^−1^, in the tranexamic acid arm (*n* = 65) of a controlled cohort of 130 patients undergoing primary total knee arthroplasty	Falls below 50 mg L^−1^ by six weeks after uncomplicated primary total knee arthroplasty; measured at weeks 2, 4 and 6 in 61 patients (32 unilateral, 29 simultaneous bilateral)	A CRP that does not follow this declining course warrants further evaluation. No threshold applicable to the first two postoperative weeks was identified in the sources reviewed here	[88,89]
ESR	Day 3: 15.41 ± 4.39 mm h^−1^, same tranexamic acid arm (*n* = 65)	Falls below 30 mm h^−1^ by six weeks after uncomplicated primary total knee arthroplasty, same cohort	Persistent elevation beyond six weeks is the deviation described. The slower kinetics of ESR limit its value early after surgery	[88,89]
IL-6	No postoperative value in primary arthroplasty was identified in the sources reviewed here	The postoperative trajectory after primary arthroplasty was not characterized in the sources reviewed here	In revision hip and knee arthroplasty (*n* = 218), serum IL-6 discriminated acute periprosthetic joint infection better than CRP (7.2 pg mL^−1^, AUC 0.929), whereas CRP performed better in chronic infection	[88,90]
Synovial white blood cell count	140 patients treated for established periprosthetic joint infection by single-stage exchange or by debridement with implant retention, combined with intra-articular antibiotic infusion; sampled every two days for 14 days	Decreased steadily from preoperative levels across the 14 days	A higher preoperative synovial white blood cell count was associated with treatment success in that cohort	[14]
Synovial PMN percentage	Same cohort and sampling schedule as above	Rose after surgery, peaked on days 1–2, then declined gradually	The decline was slower in patients whose treatment did not succeed. The authors call for larger cohorts before this pattern is relied upon	[14]

This table describes the behavior of inflammatory markers as reported in the studies cited. The populations differ between rows and are stated in each. AUC: area under the curve, CRP: C-reactive protein, ESR: erythrocyte sedimentation rate, PMN: polymorphonuclear neutrophil.

**Table 2:** Clinically used and emerging biomarkers for inflammation monitoring post arthroplasty.

Biomarker	Category	Serum (Cut-Off/Sn/Sp/AUC)	Synovial Fluid (Cut-Off/Sn/Sp/AUC)	Main Clinical Utility	Availability, Cost and Main Limitation	Ref
CRP	Acute-phase reactant	≥10 mg L^−1^ (Sn 0.794, Sp 0.777); 9.6 mg L^−1^, AUC 0.93 (Sn 0.849, Sp 0.905), thresholds stratified by sex, joint and virulence; chronic PJI 15.1 mg L^−1^, AUC 0.908 (Sn 0.774, Sp 0.918)	THA 3.00 mg L^−1^, AUC 0.90; TKA 1.65 mg L^−1^, AUC 0.88	First-line screening; predictable kinetics, peaks ~48 h; falls below 5 mg dL^−1^ by 6 weeks after uncomplicated TKA	Universally available, inexpensive. Low specificity early post-operation; suppressed by perioperative dexamethasone	[89,90,91,180,188,189]
ESR	Acute-phase reactant	>30 mm h^−1^ (Sn 0.755, Sp 0.755); 29 mm h^−1^, AUC 0.891 (Sn 0.751, Sp 0.908)	–	Screening adjunct to CRP; falls below 30 mm h^−1^ by 6 weeks after uncomplicated TKA	Universally available, inexpensive. Slow kinetics; non-specific	[89,180,188]
Fibrinogen	Acute-phase reactant	>4 g L^−1^ (Sn 0.709, Sp 0.859); 3.4 g L^−1^, AUC 0.820 (Sn 0.696, Sp 0.845); ranked second after CRP (AUC 0.917) among six routine serum markers	–	Diagnostic performance approaching CRP at lower cost	Widely available, inexpensive. Cut-offs not standardized; influenced by liver function and coagulopathies	[188,190,191]
D-dimer	Acute-phase/coagulation	>1 mg L^−1^ (Sn 0.699, Sp 0.729; serum subgroup 0.88/0.76)	236,804 ng mL^−1^, AUC 0.992 (Sn 1.00, Sp 0.941); median 927,095 vs. 20,954 ng mL^−1^, *p* < 0.001	Synovial measurement markedly outperforms serum for chronic knee PJI	Serum assay routine; synovial requires aspiration. Serum values confounded by surgery, trauma or thrombosis	[188,192]
Procalcitonin	Acute-phase reactant	>0.1 ng mL^−1^ (Sn 0.441, Sp 0.852)	–	More specific for bacterial infection than CRP;	Assay costly;	[184]
White blood cell count	Cellular	8 G L^−1^, AUC 0.71	>3000 cells µL^−1^ rule-in (Sn 0.887, Sp 0.985); >1500 cells µL^−1^ rule-out (Sn 1.00, Sp 0.88); 2318 cells µL^−1^, AUC 0.93 (Sn 0.83, Sp 0.95), EBJIS-anchored	Cornerstone of synovial evaluation; falls steadily over the first 14 postoperative days	Requires joint aspiration; inexpensive once obtained. Thresholds vary and may be confounded by inflammatory arthropathies	[14,185,193]
PMN %	Cellular	–	>75% rule-in (Sn 0.77, Sp 0.978); >65% rule-out (Sn 0.958, Sp 0.786); 64%, AUC 0.90 (Sn 0.76, Sp 0.95)	Improves specificity over total WBC; peaks on postoperative days 1–2 then declines, faster in successfully treated patients	Invasive sampling. Thresholds shift in the early postoperative period	[14,185,193]
Absolute PMN count	Cellular	–	Hip 783.6 cells µL^−1^, AUC 0.92; knee 549 cells µL^−1^, AUC 0.91; 594.2 cells µL^−1^ under ICM criteria	Joint-specific thresholds benchmarked against EBJIS and ICM 2018 criteria	Inexpensive alternative to alpha-defensin; requires aspiration	[194]
Lymphocyte %	Cellular	–	<34.7%, AUC 0.96 (Sn 0.85, Sp 0.96); decision tree with PMN count AUC 0.95 (Sn 0.93, Sp 0.91)	Outperformed alpha-defensin and synovial CRP head-to-head in 400 revisions	Derived from the routine differential count; no additional assay cost	[195]
Leukocyte esterase	Cellular/enzymatic	–	++ or ++/+ colorimetric change (Sn 0.90, Sp 0.96)	Rapid bedside rule-in test	Inexpensive dipstick. Subjective read-out; false positives due to contamination	[186]
Calprotectin	Cellular/neutrophil activation	–	~50 mg L^−1^ (Sn 0.94, Sp 0.93)	Rapid test reflecting neutrophil activation	Inexpensive and rapid but less widely available; cut-off values still debated	[196]
Alpha-defensin	Antimicrobial peptide	–	5.2 mg L^−1^ (Sn 0.878, Sp 0.979); prospective cohort cut-off 9.2, AUC 0.945 (Sn 0.921, Sp 0.981, NPV 0.998)	Very high accuracy; NPV approaching 100% supports use as a rule-out test; unaffected by prior antibiotic use	Expensive and not widely available; requires aspiration	[186,197]
IL-6	Cytokine	>10 pg mL^−1^ (Sn 0.763, Sp 0.858); acute PJI 7.2 pg mL^−1^, AUC 0.929 (Sn 0.935, Sp 0.836, NPV 0.968)	Not standardized; typical threshold ~10 pg mL^−1^ (Sn 0.87, Sp 0.90)	Rises within hours; best serum discriminator for acute PJI, whereas CRP performs better in chronic PJI	Assays costly; limited availability; short half-life	[90,181,188]
IL-1β	Cytokine	–	~313 pg mL^−1^ (Sn 0.97, Sp 0.95)	Key downstream read-out of inflammasome activation	Not routinely available; may be elevated in other inflammatory arthropathies	[198]
IL-18	Cytokine	–	359 pg mL^−1^ (Sn 1.00, Sp 1.00)	Inflammasome-related cytokine	Not routinely available; single-center derivation	[199]
IL-10	Cytokine	–	>10.305 pg mL^−1^ (Sn 0.922, Sp 0.779)	Anti-inflammatory cytokine; useful adjunct for chronic PJI diagnosis	Limited sample sizes; thresholds may differ across cohorts	[200]
HMGB1	DAMP-derived	6.8 ng mL^−1^ by ELISA (Sn 0.91, Sp 0.88)	6.8 ng mL^−1^ by ELISA	Early damage-associated molecular pattern marker; correlates with CRP and IL-6 surges	Not yet routine; limited commercial assays	[201]
Cell-free DNA	DAMP-derived	≥1.59 ng µL^−1^ (plasma)	≥1.59 ng µL^−1^ (Sn 0.90, Sp 1.00)	Detects necrotic cell death and neutrophil traps; allows pathogen identification via sequencing	Requires specialized assays and joint aspiration; limited studies	[202]
Neutrophil extracellular traps	DAMP-derived	–	cfDNA ≥9.21 µg mL^−1^ or other NET components (Sn 0.935, Sp 0.964)	Reflect innate immune activation and bacterial trapping; high accuracy in early PJI	Requires specialized assays (cfDNA, MPO, citrullinated histone H3); limited cohorts	[203]
Heat shock proteins	DAMP-derived	–	As for serum	Stress proteins acting as DAMPs	Not routinely measured; expensive ELISAs; few arthroplasty data	[47]
CRP-to-hemoglobin ratio	Composite index	AUC 0.87 (95% CI 0.85–0.90), Sn 0.81; low-virulence organisms Sn/Sp 0.83; reduced sensitivity in diabetes (0.77)	–	Helps delineate septic from aseptic inflammation	Derived from routine tests; no additional cost. Performance reduced in diabetes	[85]
CRP-to-albumin ratio/CAGR	Composite index	CAR AUC 0.87, CAGR AUC 0.87; cut-offs 2.46 and 7.09; in primary THA/TKA (*n* = 842) CAR 3.1, AUC 0.92 (Sn 0.923, Sp 0.974)	–	Among the few indices validated in a primary arthroplasty cohort; also predicts wound complications	Derived from routine tests; no additional cost	[204,205]
CRP-to-prealbumin ratio	Composite index	AUC 0.921 (95% CI 0.890–0.952), cut-off 0.0366 (Sn 0.761, Sp 0.952); diabetic subgroup AUC 0.951	–	Discriminates PJI from aseptic loosening (180 vs. 105 patients)	Derived from routine tests	[206]
PAR/CALLY index	Composite index	PAR AUC 0.779; CALLY AUC 0.647; PAR + CRP + ESR combined Sn 0.938, Sp 0.925	–	Combination outperforms any single index	Derived from routine tests; prospective multicentre validation	[207]
Glucose/serum-to-synovial ratio	Metabolic	Used as the denominator of the ratio	Synovial glucose AUC 0.861; serum-to-synovial ratio cut-off 0.69, AUC 0.889	In acute postoperative PJI, outperformed CRP (0.69), synovial WBC (0.714), and PMN % (0.66)	Inexpensive and universally available	[208]
Matrix metalloproteinases	Matrix remodeling	No validated threshold	–	Mediator of extracellular matrix remodeling; shows predictable postoperative kinetics	No validated diagnostic thresholds; evidence limited to moderate-sized cohorts	[209]

Sn = sensitivity; Sp = specificity; AUC = area under the receiver operating characteristic curve; NPV = negative predictive value; PJI = periprosthetic joint infection; THA = total hip arthroplasty; TKA = total knee arthroplasty; EBJIS = European Bone and Joint Infection Society; ICM = International Consensus Meeting; PMN = polymorphonuclear neutrophil; CAR = CRP-to-albumin ratio; CAGR = CRP-to-albumin/globulin ratio; PAR = platelet-to-albumin ratio; CALLY = C-reactive protein–albumin–lymphocyte index. Where a biomarker has been evaluated in more than one specimen type, findings are presented within a single row to permit direct comparison. “–” indicates that no usable diagnostic accuracy data were identified for that specimen. Unless otherwise indicated, diagnostic accuracy data derive from revision cohorts undergoing evaluation for suspected periprosthetic joint infection rather than from unselected primary arthroplasty populations.

## Data Availability

No new data were created or analyzed in this study. Data sharing is not applicable to this article.

## References

[1] Langkilde A., Jakobsen T.L., Bandholm T.Q., Eugen-Olsen J., Blauenfeldt T., Petersen J., Andersen O. (2017). Inflammation and post-operative recovery in patients undergoing total knee arthroplasty-secondary analysis of a randomized controlled trial. Osteoarthr. Cartil..

[2] Wickline A., Cole W., Melin M., Ehmann S., Aviles F., Bradt J. (2023). Mitigating the Post-operative Swelling Tsunami in Total Knee Arthroplasty: A Call to Action. J. Orthop. Exp. Innov..

[3] Desborough J.P. (2000). The stress response to trauma and surgery. Br. J. Anaesth..

[4] Chidambaran V., Duan Q., Pilipenko V., Glynn S.M., Sproles A., Martin L.J., Lacagnina M.J., King C.D., Ding L. (2024). The role of cytokines in acute and chronic postsurgical pain after major musculoskeletal surgeries in a quaternary pediatric center. Brain Behav. Immun..

[5] Reikeras O., Borgen P., Reseland J.E., Lyngstadaas S.P. (2014). Changes in serum cytokines in response to musculoskeletal surgical trauma. BMC Res. Notes.

[6] Chen X.-X., Wang T., Li J., Kang H. (2016). Relationship between Inflammatory Response and Estimated Complication Rate after Total Hip Arthroplasty. Chin. Med. J..

[7] Zhang H., Ma X., Chen G., Wang Z., Shang Z., Wang T., Yu T., Zhang Y. (2025). Inflammatory Marker Changes Following Total Knee Arthroplasty for Rheumatoid Arthritis with Vancomycin-Loaded Calcium Sulfate Bone Filling. J. Knee Surg..

[8] Yang L., Zhan Y.-f., Zhai Z.-j., Zhang S.-Z., Wang C.-f., Li Q., Wu B.-Y., Bian W.-W., Li H.-W., Ruan H. (2025). Inflammatory-induced swelling after total knee arthroplasty: Obesity and preoperative joint pain as key predictors. Arthroplasty.

[9] Maceška J., Navrátilová P., Goldbergová M.P. (2025). Periprosthetic inflammation: From the cellular level to clinical implications. JBMR Plus.

[10] Bitar D., Parvizi J. (2015). Biological response to prosthetic debris. World J. Orthop..

[11] Noordin S., Masri B. (2012). Periprosthetic osteolysis: Genetics, mechanisms and potential therapeutic interventions. Can. J. Surg..

[12] Khan M., Della Valle C.J., Jacofsky D.J., Meneghini R.M., Haddad F.S. (2015). Early postoperative complications after total hip arthroplasty: Current strategies for prevention and treatment. Instr. Course Lect..

[13] Bocea B.-A., Roman M.-D., Ion N.C.I., Fleaca S.R., Mohor C.-I., Popa D.A., Mihaila R.-G. (2024). Diagnostic Values of Serum Inflammatory Biomarkers after Hip and Knee Arthroplasty in Patients with Periprosthetic Joint Infection. Healthcare.

[14] Mu W., Lizcano J.D., Xu B., Li S., Zhang X., Parvizi J., Cao L. (2025). Dynamics of Synovial Fluid Markers Following Single-Stage Exchange and Debridement, Antibiotics, and Implant Retention Procedure with Topical Antibiotic Infusion in Treating Periprosthetic Joint Infection. J. Arthroplast..

[15] Shahi A., Deirmengian C., Higuera C., Chen A., Restrepo C., Zmistowski B., Parvizi J. (2015). Premature Therapeutic Antimicrobial Treatments Can Compromise the Diagnosis of Late Periprosthetic Joint Infection. Clin. Orthop. Relat. Res..

[16] Højer Karlsen A.P., Geisler A., Petersen P.L., Mathiesen O., Dahl J.B. (2015). Postoperative pain treatment after total hip arthroplasty: A systematic review. Pain.

[17] Kuchálik J., Magnuson A., Tina E., Gupta A. (2017). Does local infiltration analgesia reduce peri-operative inflammation following total hip arthroplasty? A randomized, double-blind study. BMC Anesthesiol..

[18] Soffin E.M., Gibbons M.M., Ko C.Y., Kates S.L., Wick E.C., Cannesson M., Scott M.J., Wu C.L. (2019). Evidence Review Conducted for the Agency for Healthcare Research and Quality Safety Program for Improving Surgical Care and Recovery: Focus on Anesthesiology for Total Hip Arthroplasty. Anesth. Analg..

[19] Nassar I., Fahey J., Mitchell D. (2020). Rapid recovery following hip and knee arthroplasty using local infiltration analgesia: Length of stay, rehabilitation protocol and cost savings. ANZ J. Surg..

[20] Wainwright T.W., Gill M., McDonald D.A., Middleton R.G., Reed M., Sahota O., Yates P., Ljungqvist O. (2020). Consensus statement for perioperative care in total hip replacement and total knee replacement surgery: Enhanced Recovery After Surgery (ERAS®) Society recommendations. Acta Orthop..

[21] Murvai G.F., Hozan C.T., Magheru C., Szilagyi G., Bulzan M., Murvai V.R., Cavalu S., Ghitea T.C. (2023). Highlighting the Advantages and Benefits of Non-NSAID Treatment After Total Knee Arthroplasty: A Cross-sectional Study. In Vivo.

[22] Syed I.M., Al-Rubaie S., Cohen D., Slawaska-Eng D., Al-Besher M., Khanna V. (2025). Non-Opioid Analgesics for Postoperative Pain Management Following Total Joint Arthroplasty: A Systematic Review and Meta-Analysis. J. Arthroplast..

[23] Pop A.M., Hirschmann M.T. (2025). Pharmacologic pain management strategies for reducing postoperative pain in total knee arthroplasty: A systematic review from molecular mechanisms to clinical efficiency. Arch. Orthop. Trauma Surg..

[24] Laursen C.C.W., Lunn T.H., Hägi-Pedersen D., Brorson S., Lindberg-Larsen M., Overgaard S., Jakobsen J.C., Mathiesen O. (2025). Recommended Use of Non-Steroidal Anti-Inflammatory Drugs for Pain Treatment Following Primary Total Hip and Knee Arthroplasties in Denmark. A National Survey. Acta Anaesthesiol. Scand..

[25] Wegener J.T., Kraal T., Stevens M.F., Hollmann M.W., Kerkhoffs G.M.M.J., Haverkamp D. (2016). Low-dose dexamethasone during arthroplasty: What do we know about the risks?. EFORT Open Rev..

[26] Mo Y., Wang C., Yin D., Li F. (2024). Efficacy of Dexamethasone in Reducing Pain and Inflammation and Accelerating Total Hip Arthroplasty Postoperative Recovery: A Randomized Controlled Trial. J. PeriAnesthesia Nurs..

[27] Yuenyongviwat V., Kitjakrancharoensin P., Anusitviwat C., Iamthanaporn K. (2025). Efficacy of Weight-Based Low-Dose Intravenous Dexamethasone for Pain Management Following Total Knee Arthroplasty: A Retrospective Case-Matched Study. Orthop. Rev..

[28] Goodman S.B., Konttinen Y.T., Takagi M. (2014). Joint Replacement Surgery and the Innate Immune System. J. Long-Term Eff. Med. Implants.

[29] Epsley S., Tadros S., Farid A., Kargilis D., Mehta S., Rajapakse C.S. (2021). The Effect of Inflammation on Bone. Front. Physiol..

[30] Lisowska B., Maśliński W., Małdyk P., Ząbek J., Baranowska E. (2008). The role of cytokines in inflammatory response after total knee arthroplasty in patients with rheumatoid arthritis. Rheumatol. Int..

[31] Tanaka Y., Nakayamada S., Okada Y. (2005). Osteoblasts and Osteoclasts in Bone Remodeling and Inflammation. Curr. Drug Targets-Inflamm. Allergy.

[32] Cassuto J., Folestad A., Göthlin J., Malchau H., Kärrholm J. (2018). The key role of proinflammatory cytokines, matrix proteins, RANKL/OPG and Wnt/β-catenin in bone healing of hip arthroplasty patients. Bone.

[33] Pulik Ł., Łęgosz P., Brzóska E., Mierzejewski B., Grabowska I., Ciemerych M.A., Hube R. (2025). Periprosthetic joint infection and heterotopic ossification after total hip arthroplasty: Systematic review and meta-analysis. BMC Musculoskelet. Disord..

[34] Margraf A., Ludwig N., Zarbock A., Rossaint J. (2020). Systemic Inflammatory Response Syndrome After Surgery: Mechanisms and Protection. Anesth. Analg..

[35] Gallo J., Goodman S., Konttinen Y., Raska M. (2013). Particle disease: Biologic mechanisms of periprosthetic osteolysis in total hip arthroplasty. Innate Immun..

[36] Couto M., Vasconcelos D.P., Sousa D.M., Sousa B., Conceição F., Neto E., Lamghari M., Alves C.J. (2020). The Mechanisms Underlying the Biological Response to Wear Debris in Periprosthetic Inflammation. Front. Mater..

[37] Andres B.M., Taub D.D., Gurkan I., Wenz J.F. (2003). Postoperative Fever After Total Knee Arthroplasty: The Role of Cytokines. Clin. Orthop. Relat. Res..

[38] Grosu I., Lavand’homme P., Thienpont E. (2014). Pain after knee arthroplasty: An unresolved issue. Knee Surg. Sports Traumatol. Arthrosc..

[39] Si H.-b., Yang T.-m., Zeng Y., Zhou Z.-k., Pei F.-x., Lu Y.-r., Cheng J.-q., Shen B. (2017). Correlations between inflammatory cytokines, muscle damage markers and acute postoperative pain following primary total knee arthroplasty. BMC Musculoskelet. Disord..

[40] Cocea A.-C., Stoica C.I. (2024). Interactions and Trends of Interleukins, PAI-1, CRP, and TNF-α in Inflammatory Responses during the Perioperative Period of Joint Arthroplasty: Implications for Pain Management—A Narrative Review. J. Pers. Med..

[41] Gibon E., Amanatullah D.F., Loi F., Pajarinen J., Nabeshima A., Yao Z., Hamadouche M., Goodman S.B. (2017). The biological response to orthopaedic implants for joint replacement: Part I: Metals. J. Biomed. Mater. Res. Part B Appl. Biomater..

[42] Gibon E., Córdova L.A., Lu L., Lin T.-H., Yao Z., Hamadouche M., Goodman S.B. (2017). The biological response to orthopedic implants for joint replacement. II: Polyethylene, ceramics, PMMA, and the foreign body reaction. J. Biomed. Mater. Res. Part B Appl. Biomater..

[43] Stańczyk M., van Rietbergen B. (2004). Thermal analysis of bone cement polymerisation at the cement–bone interface. J. Biomech..

[44] Tawy G.F., Rowe P.J., Riches P.E. (2016). Thermal Damage Done to Bone by Burring and Sawing With and Without Irrigation in Knee Arthroplasty. J. Arthroplast..

[45] Mayer C., Franz A., Harmsen J.-F., Queitsch F., Behringer M., Beckmann J., Krauspe R., Zilkens C. (2017). Soft-tissue damage during total knee arthroplasty: Focus on tourniquet-induced metabolic and ionic muscle impairment. J. Orthop..

[46] Tsunoda K., Sonohata M., Kugisaki H., Someya S., Honke H., Komine M., Izumi M., Ide S., Mawatari M. (2017). The Effect of Air Tourniquet on Interleukin-6 Levels in Total Knee Arthroplasty. Open Orthop. J..

[47] Lin H., Xiong W., Fu L., Yi J., Yang J. (2025). Damage-associated molecular patterns (DAMPs) in diseases: Implications for therapy. Mol. Biomed..

[48] Wu Y., Zhou J., Liu R., Zeng Y., Sun K., Li M., Peng L., Xu J., Shen B. (2022). What Is the Normal Trajectory of C-Reactive Protein, Erythrocyte Sedimentation Rate, Plasma Fibrinogen and D-Dimer after Two-Stage Exchange for Periprosthetic Joint Infection?. Orthop. Surg..

[49] Vénéreau E., Ceriotti C., Bianchi M.E. (2015). DAMPs from Cell Death to New Life. Front. Immunol..

[50] Tang J., Li M., Huang L. (2025). HMGB1 as an emerging key modulator of bone remodeling: A narrative review. Stem Cell Res. Ther..

[51] Dong C., Liu Y., Sun C., Liang H., Dai L., Shen J., Wei S., Guo S., Leong K.W., Chen Y. (2020). Identification of Specific Joint-Inflammatogenic Cell-Free DNA Molecules From Synovial Fluids of Patients With Rheumatoid Arthritis. Front. Immunol..

[52] Neupane P., Bhuju S., Thapa N., Bhattarai H.K. (2019). ATP Synthase: Structure, Function and Inhibition. Biomol. Concepts.

[53] Di Virgilio F., Sarti A.C., Coutinho-Silva R. (2020). Purinergic signaling, DAMPs, and inflammation. Am. J. Physiol. Cell. Physiol..

[54] Land W.G. (2012). Role of heat shock protein 70 in innate alloimmunity. Front. Immunol..

[55] Colaco C.A., Bailey C.R., Walker K.B., Keeble J. (2013). Heat Shock Proteins: Stimulators of Innate and Acquired Immunity. BioMed Res. Int..

[56] Andocs G., Meggyeshazi N., Balogh L., Spisak S., Maros M.E., Balla P., Kiszner G., Teleki I., Kovago C., Krenacs T. (2015). Upregulation of heat shock proteins and the promotion of damage-associated molecular pattern signals in a colorectal cancer model by modulated electrohyperthermia. Cell Stress Chaperones.

[57] Khandia R., K Munjal A., MN Iqbal H., Dhama K. (2016). Heat shock proteins: Therapeutic perspectives in inflammatory disorders. Recent Pat. Inflamm. Allergy Drug Discov..

[58] Szatmary P., Huang W., Criddle D., Tepikin A., Sutton R. (2018). Biology, role and therapeutic potential of circulating histones in acute inflammatory disorders. J. Cell. Mol. Med..

[59] Li X., Ye Y., Peng K., Zeng Z., Chen L., Zeng Y. (2022). Histones: The critical players in innate immunity. Front. Immunol..

[60] Richards C.M., McRae S.A., Ranger A.L., Klegeris A. (2023). Extracellular histones as damage-associated molecular patterns in neuroinflammatory responses. Rev. Neurosci..

[61] Li D., Wu M. (2021). Pattern recognition receptors in health and diseases. Signal Transduct. Target. Ther..

[62] Netea M.G., van de Veerdonk F.L., Kullberg B.J., Van der Meer J.W., Joosten L.A. (2008). The role of NLRs and TLRs in the activation of the inflammasome. Expert Opin. Biol. Ther..

[63] Zhou G., Liu M., Wu X., Tang B. (2023). Dynamic changes and clinic significance of serum high mobility group box 1 in patients with total hip arthroplasty. Cir. Y Cir..

[64] Zou Y., Wu Y., Wei A., Nie H., Hui S., Liu C., Deng T. (2025). Serum HMGB1 as a predictor for postoperative delirium in elderly patients undergoing total hip arthroplasty surgery. Adv. Clin. Exp. Med..

[65] Cobra H.A.d.A.B., Mozella A.P., da Palma I.M., Salim R., Leal A.C. (2022). Cell-free Deoxyribonucleic Acid: A Potential Biomarker of Chronic Periprosthetic Knee Joint Infection. J. Arthroplast..

[66] de Sandes Kimura O., Mozella A., Cobra H., Maciel Saraiva A.C., Carvalho de Almendra Freitas E.H., Cury Fernandes M.B., Matheus Guimarães J.A., Defino H., Leal A.C. (2024). Neutrophil Extracellular Trap-related Biomarkers Are Increased in the Synovial Fluid of Patients With Periprosthetic Joint Infections. Clin. Orthop. Relat. Res..

[67] Xu J., Núñez G. (2023). The NLRP3 inflammasome: Activation and regulation. Trends Biochem. Sci..

[68] Huang D., Jiang C., Ren H., Wang J., Li S., Song W., Lin Z., Wu Z., Ke X. (2025). Perioperative Repetitive Transcranial Magnetic Stimulation Reduces Postoperative Cognitive Dysfunction and Inflammation in Elderly Patients Undergoing Total Knee Arthroplasty: A Randomized Controlled Trial. J. Arthroplast..

[69] Liu X., Hu X., Li R., Zhang Y. (2020). Combination of post-fascia iliaca compartment block and dexmedetomidine in pain and inflammation control after total hip arthroplasty for elder patients: A randomized control study. J. Orthop. Surg. Res..

[70] Lee S. (2019). Dexmedetomidine: Present and future directions. Korean J. Anesthesiol..

[71] Konishi S., Narita T., Hatakeyama S., Yoneyama T., Yoneyama M.S., Tobisawa Y., Noro D., Sato T., Togashi K., Okamoto T. (2021). Utility of total cell-free DNA levels for surgical damage evaluation in patients with urological surgeries. Sci. Rep..

[72] Tatani I., Kalaitzopoulou E., Skipitari M., Ntoukas A., Tsaliki E.A., Giakoumakis S., Lakoumentas J., Varemmenou A., Michail E., Papadea P. (2025). Reduction of oxidative stress in total knee arthroplasty using tourniquet with a novel pharmaceutical combination. SICOT-J.

[73] Tenório M.C.d.S., Graciliano N.G., Moura F.A., de Oliveira A.C.M., Goulart M.O.F. (2021). N-Acetylcysteine (NAC): Impacts on Human Health. Antioxidants.

[74] Sato H., Goto W., Yamamura J.-I., Kurokawa M., Kageyama S., Takahara T., Watanabe A., Shiraki K. (1996). Therapeutic basis of glycyrrhizin on chronic hepatitis B. Antivir. Res..

[75] Cinatl J., Morgenstern B., Bauer G., Chandra P., Rabenau H., Doerr H.W. (2003). Glycyrrhizin, an active component of liquorice roots, and replication of SARS-associated coronavirus. Lancet.

[76] Chen R.-Y., Shi J.-J., Liu Y.-J., Yu J., Li C.-Y., Tao F., Cao J.-F., Yang G.-J., Chen J., Chen R.-Y. (2024). The State-of-the-Art Antibacterial Activities of Glycyrrhizin: A Comprehensive Review. Microorganisms.

[77] Zhou S., Liu G., Si Z., Yu L., Hou L. (2021). Glycyrrhizin, an HMGB1 inhibitor, Suppresses Interleukin-1β-Induced Inflammatory Responses in Chondrocytes from Patients with Osteoarthritis. Cartilage.

[78] Yamada C., Ho A., Akkaoui J., Garcia C., Duarte C., Movila A. (2021). Glycyrrhizin mitigates inflammatory bone loss and promotes expression of senescence-protective sirtuins in an aging mouse model of periprosthetic osteolysis. Biomed. Pharmacother..

[79] Yoshihara-Hirata C., Yamashiro K., Yamamoto T., Aoyagi H., Ideguchi H., Kawamura M., Suzuki R., Ono M., Wake H., Nishibori M. (2018). Anti-HMGB1 Neutralizing Antibody Attenuates Periodontal Inflammation and Bone Resorption in a Murine Periodontitis Model. Infect. Immun..

[80] Lauková L., Konečná B., Janovičová Ľ., Vlková B., Celec P. (2020). Deoxyribonucleases and Their Applications in Biomedicine. Biomolecules.

[81] Janovičová Ľ., Kmeťová K., Pribulová N., Janko J., Gromová B., Gardlík R., Celec P. (2023). Endogenous DNase Activity in an Animal Model of Acute Liver Failure. Int. J. Mol. Sci..

[82] Weber C., Jenke A., Chobanova V., Yazdanyar M., Chekhoeva A., Eghbalzadeh K., Lichtenberg A., Wahlers T., Akhyari P., Paunel-Görgülü A. (2019). Targeting of cell-free DNA by DNase I diminishes endothelial dysfunction and inflammation in a rat model of cardiopulmonary bypass. Sci. Rep..

[83] Chen X.-Q., Tu L., Tang Q., Zou J.-S., Yun X., Qin Y.-H. (2023). DNase I targeted degradation of neutrophil extracellular traps to reduce the damage on IgAV rat. PLoS ONE.

[84] Shi C., Dawulieti J., Shi F., Yang C., Qin Q., Shi T., Wang L., Hu H., Sun M., Ren L. (2022). A nanoparticulate dual scavenger for targeted therapy of inflammatory bowel disease. Sci. Adv..

[85] Aimaiti A., Guo W., Xu B., Mu W., Wahafu T., Zou C., Hua L., Cao L. (2025). Serum C-Reactive Protein to Hemoglobin Ratio: Novel Biomarkers for the Diagnosis of Chronic Periprosthetic Joint Infection. J. Arthroplast..

[86] Benson M., Denyer S., Wozniak A., Schmitt D., Brown N. (2025). Results of Aspiration, Erythrocyte Sedimentation Rate, and C-Reactive Protein in Patients with Known Prosthetic Joint Infection on Chronic Suppression. J. Am. Acad. Orthop. Surg. Glob. Res. Rev..

[87] Yu B.-Z., Li R., Li X., Chai W., Zhou Y.-G., Chen J.-Y. (2021). The relationship of C-reactive protein/interleukin-6 concentrations between serum and synovial fluid in the diagnosis of periprosthetic joint infection. J. Orthop. Surg. Res..

[88] Lin S., Zhang X., Xia X., Xu G., Pan H. (2025). Study on the Effects of Tranexamic Acid on Perioperative Inflammation, Long-Term Functional Recovery, and Satisfaction in Total Knee Arthroplasty: A Follow-up Study. J. Inflamm. Res..

[89] Incesoy M.A., Demirkiran C.B., Kaya H.B., Geckalan M.A., Tak A.Y., Elmali N., Yildiz F., Uzer G. (2025). Natural course of postoperative C-reactive protein and erythrocyte sedimentation rate in unilateral and simultaneous bilateral total knee arthroplasty. BMC Musculoskelet. Disord..

[90] Zhang X., Lu F., Zhang B., Liu Z., Geng B., Han H., Xia Y. (2025). Serum Interleukin-6 Exhibits Better Diagnostic Performance Than Serum C-Reactive Protein in Acute Periprosthetic Joint Infection. J. Arthroplast..

[91] Chen J., He Y., Deng T., Li Y., Wang X., Zhao M., Li F., Wang C., Tian H. (2025). Effects of Dexamethasone on Nausea, Vomiting, and Inflammatory Indexes After Total Knee Arthroplasty. Orthop. Surg..

[92] Mahmoud Issa L., Kehlet H., Madsbad S., Lindberg-Larsen M., Varnum C., Jakobsen T., Andersen M.R., Bieder M.J., Overgaard S., Hansen T.B. (2025). Perioperative High-Dose Steroid in Insulin-Treated Patients With Diabetes Undergoing Fast-Track Hip and Knee Arthroplasty: Impact on Length of Stay and Discharge Blood Glucose Levels. Acta Anaesthesiol. Scand..

[93] Lewis K., Alshamsi F., Carayannopoulos K.L., Granholm A., Piticaru J., Al Duhailib Z., Chaudhuri D., Spatafora L., Yuan Y., Centofanti J. (2022). Dexmedetomidine vs other sedatives in critically ill mechanically ventilated adults: A systematic review and meta-analysis of randomized trials. Intensive Care Med..

[94] Deng L., Tan L. (2022). Effects of Parecoxib Sodium Application Combined with Enhanced Recovery After Surgery Nursing on Inflammatory Factors and Knee Joint Function in Elderly Patients After Total Knee Arthroplasty. Front. Surg..

[95] Wang D., Luo Z.Y., Yu Z.P., Liu L.X., Chen C., Meng W.K., Yu Q.P., Pei F.X., Zhou Z.K., Zeng W.N. (2018). The antifibrinolytic and anti-inflammatory effects of multiple doses of oral tranexamic acid in total knee arthroplasty patients: A randomized controlled trial. J. Thromb. Haemost..

[96] Zhang S., Xu H., Xie J., Cao G., Lei Y., Pei F. (2020). Tranexamic acid attenuates inflammatory effect and modulates immune response in primary total knee arthroplasty: A randomized, placebo-controlled, pilot trial. Inflammopharmacology.

[97] Okholm S.H., Krog J., Hvas A.-M. (2022). Tranexamic Acid and Its Potential Anti-Inflammatory Effect: A Systematic Review. Semin. Thromb. Hemost..

[98] Ma K., Wu X. (2025). Effects of tranexamic acid on postoperative knee activity and stress, and inflammation cytokines in patients undergoing total knee arthroplasty. Inflammopharmacology.

[99] Poeran J., Rasul R., Suzuki S., Danninger T., Mazumdar M., Opperer M., Boettner F., Memtsoudis S.G. (2014). Tranexamic acid use and postoperative outcomes in patients undergoing total hip or knee arthroplasty in the United States: Retrospective analysis of effectiveness and safety. BMJ.

[100] Yi M., Li T., Niu M., Zhang H., Wu Y., Wu K., Dai Z. (2024). Targeting cytokine and chemokine signaling pathways for cancer therapy. Signal Transduct. Target. Ther..

[101] Yaszay B., Trindade M.C.D., Lind M., Goodman S.B., Smith R.L. (2001). Fibroblast expression of C—C chemokines in response to orthopaedic biomaterial particle challenge in vitro. J. Orthop. Res..

[102] Bastian D., Tamburstuen M.V., Lyngstadaas S.P., Reikerås O. (2008). Systemic and Local Cytokine Kinetics after Total Hip Replacement Surgery. Eur. Surg. Res..

[103] Hallab N.J., Jacobs J.J. (2017). Chemokines Associated with Pathologic Responses to Orthopedic Implant Debris. Front. Endocrinol..

[104] O’Shea J.J., Murray P.J. (2008). Cytokine Signaling Modules in Inflammatory Responses. Immunity.

[105] Ott N., Faletti L., Heeg M., Andreani V., Grimbacher B. (2023). JAKs and STATs from a Clinical Perspective: Loss-of-Function Mutations, Gain-of-Function Mutations, and Their Multidimensional Consequences. J. Clin. Immunol..

[106] Liu T., Zhang L., Joo D., Sun S.-C. (2017). NF-κB signaling in inflammation. Signal Transduct. Target. Ther..

[107] Chapnick D.A., Warner L., Bernet J., Rao T., Liu X. (2011). Partners in crime: The TGFβ and MAPK pathways in cancer progression. Cell Biosci..

[108] Braicu C., Buse M., Busuioc C., Drula R., Gulei D., Raduly L., Rusu A., Irimie A., Atanasov A.G., Slaby O. (2019). A Comprehensive Review on MAPK: A Promising Therapeutic Target in Cancer. Cancers.

[109] Cargnello M., Roux P.P. (2011). Activation and Function of the MAPKs and Their Substrates, the MAPK-Activated Protein Kinases. Microbiol. Mol. Biol. Rev. MMBR.

[110] Qoreishi M., Panahi M., Dorodi O., Ghanbari N., Jousheghan S.S. (2022). Involvement of NF-κB/NLRP3 axis in the progression of aseptic loosening of total joint arthroplasties: A review of molecular mechanisms. Naunyn-Schmiedeberg’s Arch. Pharmacol..

[111] Tas S.W., Vervoordeldonk M.J., Hajji N., May M.J., Ghosh S., Tak P.P. (2006). Local treatment with the selective IκB kinase β inhibitor NEMO-binding domain peptide ameliorates synovial inflammation. Arthritis Res. Ther..

[112] Strickland I., Ghosh S. (2006). Use of cell permeable NBD peptides for suppression of inflammation. Ann. Rheum. Dis..

[113] Wang Y., Zhang C., Xu W., Wang B., Lan Y., Yu M., Wang P., Xie Z. (2018). The effect of surface immobilized NBD peptide on osteoclastogenesis of rough titanium plates in vitro and osseointegration of rough titanium implants in ovariectomized rats in vivo. RSC Adv..

[114] Lan Y., Xie H., Shi Y., Jin Q., Zhang X., Wang Y., Xie Z. (2019). NEMO-binding domain peptide ameliorates inflammatory bone destruction in a Staphylococcus aureus-induced chronic osteomyelitis model. Mol. Med. Rep..

[115] Glaeser J.D., Salehi K., Kanim L.E., NaPier Z., Kropf M.A., Cuéllar J.M., Perry T.G., Bae H.W., Sheyn D. (2020). NF-κB inhibitor, NEMO-binding domain peptide attenuates intervertebral disc degeneration. Spine J..

[116] Ren X., Li X., Jia L., Chen D., Hou H., Rui L., Zhao Y., Chen Z. (2017). A small-molecule inhibitor of NF-κB-inducing kinase (NIK) protects liver from toxin-induced inflammation, oxidative stress, and injury. FASEB J..

[117] Ren W., Wu B., Peng X., Mayton L., Yu D., Ren J., Chen B.D., Wooley P.H. (2006). Erythromycin inhibits wear debris-induced inflammatory osteolysis in a murine model. J. Orthop. Res..

[118] Chen D., Zhang X., Guo Y., Shi S., Mao X., Pan X., Cheng T. (2012). MMP-9 inhibition suppresses wear debris-induced inflammatory osteolysis through downregulation of RANK/RANKL in a murine osteolysis model. Int. J. Mol. Med..

[119] Tordjman S., Chokron S., Delorme R., Charrier A., Bellissant E., Jaafari N., Fougerou C. (2017). Melatonin: Pharmacology, Functions and Therapeutic Benefits. Curr. Neuropharmacol..

[120] Ping Z., Hu X., Wang L., Shi J., Tao Y., Wu X., Hou Z., Guo X., Zhang W., Yang H. (2017). Melatonin attenuates titanium particle-induced osteolysis via activation of Wnt/β-catenin signaling pathway. Acta Biomater..

[121] Wu Y., He F., Zhang C., Zhang Q., Su X., Zhu X., Liu A., Shi W., Lin W., Jin Z. (2021). Melatonin alleviates titanium nanoparticles induced osteolysis via activation of butyrate/GPR109A signaling pathway. J. Nanobiotechnol..

[122] Frost H.M. (2004). A 2003 update of bone physiology and Wolff’s Law for clinicians. Angle Orthod..

[123] Boyle W.J., Simonet W.S., Lacey D.L. (2003). Osteoclast differentiation and activation. Nature.

[124] Alesi D., Zinno R., Scoppolini Massini M., Barone G., Valente D., Pinelli E., Zaffagnini S., Mirulla A.I., Bragonzoni L. (2025). Variations in bone mineral density after joint replacement: A systematic review examining different anatomical regions, fixation techniques and implant design. J. Exp. Orthop..

[125] Matsuo K., Galson D.L., Zhao C., Peng L., Laplace C., Wang K.Z.Q., Bachler M.A., Amano H., Aburatani H., Ishikawa H. (2004). Nuclear factor of activated T-cells (NFAT) rescues osteoclastogenesis in precursors lacking c-Fos. J. Biol. Chem..

[126] Nakashima T., Takayanagi H. (2009). Osteoimmunology: Crosstalk between the immune and bone systems. J. Clin. Immunol..

[127] Goodman S.B., Gibon E., Gallo J., Takagi M. (2022). Macrophage Polarization and the Osteoimmunology of Periprosthetic Osteolysis. Curr. Osteoporos. Rep..

[128] Toita R., Kang J.H., Tsuchiya A. (2022). Phosphatidylserine liposome multilayers mediate the M1-to-M2 macrophage polarization to enhance bone tissue regeneration. Acta Biomater..

[129] Gao Q., Rhee C., Maruyama M., Li Z., Shen H., Zhang N., Utsunomiya T., Huang E.E., Yao Z., Bunnell B.A. (2021). The Effects of Macrophage Phenotype on Osteogenic Differentiation of MSCs in the Presence of Polyethylene Particles. Biomedicines.

[130] Zhao D.W., Ren B., Wang H.W., Zhang X., Yu M.Z., Cheng L., Sang Y.H., Cao S.S., Thieringer F.M., Zhang D. (2021). 3D-printed titanium implant combined with interleukin 4 regulates ordered macrophage polarization to promote bone regeneration and angiogenesis. Bone Jt. Res..

[131] Mahon O.R., O’Hanlon S., Cunningham C.C., McCarthy G.M., Hobbs C., Nicolosi V., Kelly D.J., Dunne A. (2018). Orthopaedic implant materials drive M1 macrophage polarization in a spleen tyrosine kinase- and mitogen-activated protein kinase-dependent manner. Acta Biomater..

[132] Bijukumar D.R., Salunkhe S., Zheng G., Barba M., Hall D.J., Pourzal R., Mathew M.T. (2020). Wear particles induce a new macrophage phenotype with the potential to accelerate material corrosion within total hip replacement interfaces. Acta Biomater..

[133] Ge Y.W., Feng K., Liu X.L., Zhu Z.A., Chen H.F., Chang Y.Y., Sun Z.Y., Wang H.W., Zhang J.W., Yu D.G. (2020). Quercetin inhibits macrophage polarization through the p-38α/β signalling pathway and regulates OPG/RANKL balance in a mouse skull model. J. Cell. Mol. Med..

[134] Pajarinen J., Lin T., Nabeshima A., Sato T., Gibon E., Jämsen E., Khan T.N., Yao Z., Goodman S.B. (2021). Interleukin-4 repairs wear particle induced osteolysis by modulating macrophage polarization and bone turnover. J. Biomed. Mater. Res. A.

[135] de Molon R.S., Tetradis S., Vernal R., Leite F.R.M., Van Dyke T.E. (2026). Periodontitis as a Model for Inflammatory Uncoupling of Bone Remodeling. J. Periodontal. Res..

[136] Roato I., Caldo D., D’Amico L., D’Amelio P., Godio L., Patanè S., Astore F., Grappiolo G., Boggio M., Scagnelli R. (2010). Osteoclastogenesis in peripheral blood mononuclear cell cultures of periprosthetic osteolysis patients and the phenotype of T cells localized in periprosthetic tissues. Biomaterials.

[137] Ishida T., Tateiwa T., Takahashi Y., Takahashi R.H., Sano K., Shishido T., Masaoka T., Yamamoto K. (2021). IL-17A-Mediated Immune-Inflammatory Periarticular Mass and Osteolysis From Impingement in Ceramic-On-Ceramic Total Hip Arthroplasty. Arthroplast. Today.

[138] Durdan M.M., Azaria R.D., Weivoda M.M. (2022). Novel insights into the coupling of osteoclasts and resorption to bone formation. Semin. Cell Dev. Biol..

[139] Arthur A., Gronthos S. (2021). Eph-Ephrin Signaling Mediates Cross-Talk Within the Bone Microenvironment. Front. Cell Dev. Biol..

[140] Matsuo K., Otaki N. (2012). Bone cell interactions through Eph/ephrin: Bone modeling, remodeling and associated diseases. Cell Adh. Migr..

[141] Grewe J.M., Knapstein P.R., Donat A., Jiang S., Smit D.J., Xie W., Keller J. (2022). The role of sphingosine-1-phosphate in bone remodeling and osteoporosis. Bone Res..

[142] Qiu H., Liu S., Jiang Y., Lai Y., Lin Q., Huang A. (2026). Beyond resorption-driven coupling: A multi-layered framework for osteoclast-osteoblast communication and its therapeutic consequences. Front. Endocrinol..

[143] Reikerås O., Shegarfi H., Wang J.E., Utvåg S.E. (2005). Lipopolysaccharide impairs fracture healing: An experimental study in rats. Acta Orthop..

[144] Toben D., Schroeder I., El Khassawna T., Mehta M., Hoffmann J.-E., Frisch J.-T., Schell H., Lienau J., Serra A., Radbruch A. (2011). Fracture healing is accelerated in the absence of the adaptive immune system. J. Bone Miner. Res. Off. J. Am. Soc. Bone Miner. Res..

[145] Pentland V., Thompson Z., Dayimu A., Demiris N., Bohm E., Campbell D., Lenguerrand E., Fenstad A.M., Furnes O.N., Hailer N. (2026). Survivorship of modern total hip replacement to 30 years: Systematic review, meta-analysis, and extrapolation of global joint registry data. Lancet.

[146] Gausden E.B., Puri S., Chiu Y.F., Figgie M.P., Sculco T.P., Westrich G., Sculco P.K., Chalmers B.P. (2023). Mid-term survivorship of primary total knee arthroplasty with a specific implant. Bone Jt. J..

[147] Hasan S., Marang-van de Mheen P.J., Kaptein B.L., Nelissen R., Pijls B.G. (2020). RSA-tested TKA Implants on Average Have Lower Mean 10-year Revision Rates Than Non-RSA-tested Designs. Clin. Orthop. Relat. Res..

[148] Baek J.H., Lee S.C., Choi K., Ahn H.S., Nam C.H. (2021). Long-term survivorship of total knee arthroplasty with a single-radius, high-flexion posterior stabilized prosthesis. Knee.

[149] Lin Y., Gao J., Zheng H., Zhang Z., Sun T. (2025). Perioperative Use of Bisphosphonate and Implant Survival After Total Joint Arthroplasty. J. Arthroplast..

[150] Wheatley B.M., Nappo K.E., Christensen D.L., Holman A.M., Brooks D.I., Potter B.K. (2019). Effect of NSAIDs on Bone Healing Rates: A Meta-analysis. J. Am. Acad. Orthop. Surg..

[151] Friedrich M.J., Wimmer M.D., Schmolders J., Strauss A.C., Ploeger M.M., Kohlhof H., Wirtz D.C., Gravius S., Randau T.M. (2017). RANK-ligand and osteoprotegerin as biomarkers in the differentiation between periprosthetic joint infection and aseptic prosthesis loosening. World J. Orthop..

[152] Xu J., Li H., Qu Y., Zheng C., Wang B., Shen P., Xie Z., Wei K., Wang Y., Zhao J. (2021). Denosumab might prevent periprosthetic bone loss after total hip and knee arthroplasties: A review. Arthroplasty.

[153] Murahashi Y., Teramoto A., Jimbo S., Okada Y., Kamiya T., Imamura R., Takashima H., Watanabe K., Nagoya S., Yamashita T. (2020). Denosumab prevents periprosthetic bone mineral density loss in the tibial metaphysis in total knee arthroplasty. Knee.

[154] Goodman S.B., Gallo J. (2019). Periprosthetic Osteolysis: Mechanisms, Prevention and Treatment. J. Clin. Med..

[155] Reginster J.-Y. (2014). Cardiac concerns associated with strontium ranelate. Expert Opin. Drug Saf..

[156] Sköld C., Kultima K., Freyhult E., Larsson A., Gordh T., Hailer N.P., Mallmin H. (2022). Effects of denosumab treatment on the expression of receptor activator of nuclear kappa-B ligand (RANKL) and TNF-receptor TNFRSF9 after total hip arthroplasty—Results from a randomized placebo-controlled clinical trial. Osteoporos. Int..

[157] Jang G., Kaufman A., Lee E., Hamilton L., Hutton S., Egbuna O., Padhi D. (2014). A clinical therapeutic protein drug–drug interaction study: Coadministration of denosumab and midazolam in postmenopausal women with osteoporosis. Pharmacol. Res. Perspect..

[158] Akira S., Uematsu S., Takeuchi O. (2006). Pathogen Recognition and Innate Immunity. Cell.

[159] Tang D., Kang R., Coyne C.B., Zeh H.J., Lotze M.T. (2012). PAMPs and DAMPs: Signal 0s that spur autophagy and immunity. Immunol. Rev..

[160] Nie J., Zhou L., Tian W., Liu X., Yang L., Yang X., Zhang Y., Wei S., Wang D.W., Wei J. (2025). Deep insight into cytokine storm: From pathogenesis to treatment. Signal Transduct. Target. Ther..

[161] Soares C.L.R., Wilairatana P., Silva L.R., Moreira P.S., Vilar Barbosa N.M.M., da Silva P.R., Coutinho H.D.M., de Menezes I.R.A., Felipe C.F.B. (2023). Biochemical aspects of the inflammatory process: A narrative review. Biomed. Pharmacother..

[162] Saavedra-Torres J.S., Pinzon-Fernandez M.V., Ocampo-Posada M., Nati-Castillo H.A., Jimenez Hincapie L.A., Cadrazo-Gil E.J., Arias-Intriago M., Rojas-Cadena M., Tello-De-la-Torre A., Osejos W. (2025). Inflammasomes and Signaling Pathways: Key Mechanisms in the Pathophysiology of Sepsis. Cells.

[163] Brubaker S.W., Bonham K.S., Zanoni I., Kagan J.C. (2015). Innate Immune Pattern Recognition: A Cell Biological Perspective. Annu. Rev. Immunol..

[164] Wänström J.E., Dettmer A., Björnsson Hallgren H.C., Salomonsson B., Ljungquist O., Adolfsson L.E. (2025). Antibiotic prophylaxis and incidence of infection following elbow arthroplasty: A nationwide study. Acta Orthop..

[165] Salar O., Phillips J., Porter R. (2021). Diagnosis of knee prosthetic joint infection; aspiration and biopsy. Knee.

[166] Beam E., Osmon D. (2018). Prosthetic Joint Infection Update. Infect. Dis. Clin. N. Am..

[167] Wagenaar F.-C.B.M., Löwik C.A.M., Zahar A., Jutte P.C., Gehrke T., Parvizi J. (2019). Persistent Wound Drainage After Total Joint Arthroplasty: A Narrative Review. J. Arthroplast..

[168] Ayoade F., Li D., Mabrouk A., Todd J.R. (2025). Periprosthetic Joint Infection. StatPearls.

[169] Zhang J., Zhang X.-Y., Jiang F.-L., Wu Y.-P., Yang B.-B., Liu Z.-Y., Liu D. (2019). Antibiotic-impregnated bone cement for preventing infection in patients receiving primary total hip and knee arthroplasty: A meta-analysis. Medicine.

[170] Yu M., Wei Z., Yang X., Xu Y., Zhu W., Weng X., Feng B. (2024). Safety and effectiveness of intraosseous regional prophylactic antibiotics in total knee arthroplasty: A systematic review and meta-analysis. Arch. Orthop. Trauma Surg..

[171] Lee S., Kang J., Moon Y., Hong J., Kim H., Jo S. (2025). Efficacy and Safety of Intraosseous Versus Intravenous Antibiotic in Primary and Revision Total Joint Arthroplasty: A Systematic Review and Meta-Analysis. Medicina.

[172] Ma T., Lyu J., Ma J., Huang X., Chen K., Wang S., Wei Y., Shi J., Xia J., Zhao G. (2023). Comparative analysis of pathogen distribution in patients with fracture-related infection and periprosthetic joint infection: A retrospective study. BMC Musculoskelet. Disord..

[173] Nieboer M., Braig Z., Rosenow C., Marigi E., Tande A., Barlow J., Sanchez-Sotelo J., O’Driscoll S., Morrey M. (2024). Non-cefazolin antibiotic prophylaxis is associated with higher rates of elbow periprosthetic joint infection. J. Shoulder Elb. Surg..

[174] Graif N., Amzallag N., Kadar A., Ashkenazi I., Factor S., Gold A., Snir N., Warschawski Y. (2025). Increased rates of periprosthetic joint infection following hip hemiarthroplasty with clindamycin prophylaxis compared to cefazolin. Arch. Orthop. Trauma Surg..

[175] Ribau A.I., Collins J.E., Chen A.F., Sousa R.J. (2021). Is Preoperative Staphylococcus aureus Screening and Decolonization Effective at Reducing Surgical Site Infection in Patients Undergoing Orthopedic Surgery? A Systematic Review and Meta-Analysis With a Special Focus on Elective Total Joint Arthroplasty. J. Arthroplast..

[176] Slullitel P.A., Oñativia J.I., Buttaro M.A., Sánchez M.L., Comba F., Zanotti G., Piccaluga F. (2018). State-of-the-art diagnosis and surgical treatment of acute peri-prosthetic joint infection following primary total hip arthroplasty. EFORT Open Rev..

[177] Dudareva M., Barrett L., Figtree M., Scarborough M., Watanabe M., Newnham R., Wallis R., Oakley S., Kendrick B., Stubbs D. (2018). Sonication versus Tissue Sampling for Diagnosis of Prosthetic Joint and Other Orthopedic Device-Related Infections. J. Clin. Microbiol..

[178] Sigmund I.K., Luger M., Windhager R., McNally M.A. (2022). Diagnosing periprosthetic joint infections: A comparison of infection definitions: EBJIS 2021, ICM 2018, and IDSA 2013. Bone Jt. Res..

[179] Geno Tai D.B., Patel R., Abdel M.P., Berbari E.F., Tande A.J. (2022). Microbiology of Hip and Knee Periprosthetic Joint Infections: A Database Study. Clin. Microbiol. Infect. Off. Publ. Eur. Soc. Clin. Microbiol. Infect. Dis..

[180] Grzelecki D., Kocon M., Mazur R., Grajek A., Kowalczewski J. (2025). The diagnostic accuracy of blood C-reactive protein and erythrocyte sedimentation rate in periprosthetic joint infections—A 10-year analysis of 1510 revision hip and knee arthroplasties from a single orthopaedic center. J. Orthop. Surg. Res..

[181] Li J., Zhou Q., Deng B. (2022). Serum versus synovial fluid interleukin-6 for periprosthetic joint infection diagnosis: A systematic review and meta-analysis of 30 diagnostic test accuracy studies. J. Orthop. Surg. Res..

[182] Yang F., Zhao C., Huang R., Ma H., Wang X., Wang G., Zhao X. (2021). Plasma fibrinogen in the diagnosis of periprosthetic joint infection. Sci. Rep..

[183] Lu G., Li T., Ye H., Liu S., Zhang P., Wang W. (2020). D-dimer in the diagnosis of periprosthetic joint infection: A systematic review and meta-analysis. J. Orthop. Surg. Res..

[184] Sun X., Li Y., Lv Y., Liu Y., Lai Z., Zeng Y., Zhang H. (2024). Diagnostic value of procalcitonin in patients with periprosthetic joint infection: A diagnostic meta-analysis. Front. Surg..

[185] Sabater-Martos M., Clauss M., Ribau A., Sousa R. (2025). Differential synovial fluid white blood cell count for the diagnosis of chronic peri-prosthetic joint infection—A systematic review and meta-analysis. J. Bone Jt. Infect..

[186] Li Z., Zhang Q., Shi L., Gao F., Sun W., Li Z. (2020). Alpha-Defensin versus Leukocyte Esterase in Periprosthetic Joint Infection: An Updated Meta-Analysis. BioMed Res. Int..

[187] Hantouly A.T., Salameh M., Toubasi A.A., Salman L.A., Alzobi O., Ahmed A.F., Hameed S., Zikria B., Ahmed G. (2022). Synovial fluid calprotectin in diagnosing periprosthetic joint infection: A meta-analysis. Int. Orthop..

[188] Sigmund I.K., Puchner S.E., Windhager R. (2021). Serum inflammatory markers for the screening and diagnosis of periprosthetic joint infection: A systematic review and meta-analysis. Biomedicines.

[189] Sebastian S., Mitterer J.A., Ahmed Y., Frank B.J.H., Simon S., Hofstaetter J.G. (2025). Clinical Benefit of Using Differential Cutoff Values of Synovial C-Reactive Protein in Acute and Chronic Infected as Well as Aseptic Hip and Knee Revision Arthroplasties. J. Arthroplast..

[190] Sun Z., Zhang Q., Ma H., Wang X., Hua C., Yang F. (2025). Assessment of serum diagnostic biomarkers for periprosthetic joint infection in hip and knee arthroplasty: A retrospective study. PeerJ.

[191] Zhu G.Y., Ma C., Liu G.W., Man J.Z. (2025). [Preoperative diagnostic efficacy of novel blood markers white blood cell ratio and fibrinogen levels in periprosthetic joint infection]. Zhongguo GU Shang.

[192] Güneş Z., Yılmaz M.K., Kemah B., Çağlar Ö., Tokgözoğlu A.M., Parvizi J., Azboy İ., Atilla B. (2025). Synovial D-dimer is a novel and accurate test for diagnosis of chronic knee periprosthetic joint infection. Knee.

[193] Donner S., Matziolis G., Gramlich Y., Lazic I., Schrednitzki D., Pohrt A., Renz N., Meißner N. (2026). Lower synovial leucocyte count and polymorphonuclear percentage reliably differentiate periprosthetic joint infection after unicompartmental knee arthroplasty. Knee Surg. Sports Traumatol. Arthrosc..

[194] Sebastian S., Mitterer J.A., Ahmed Y., Frank B.J.H., Simon S., Hofstaetter J.G. (2025). Synovial absolute polymorphonuclear neutrophil cell count: A simple and inexpensive marker for diagnosing periprosthetic hip and knee joint infections. Knee Surg. Sports Traumatol. Arthrosc..

[195] Alimy A.R., Sijakov J., Jandl N.M., Bartsch P., Ries C., Hubert J., Tolosa E., Rohde H., Keller J., Beil F.T. (2025). Low Synovial Lymphocyte Percentage as a Novel Diagnostic Marker of Periprosthetic Joint Infection. JB JS Open Access.

[196] Peng X., Zhang H., Xin P., Bai G., Ge Y., Cai M., Wang R., Fan Y., Pang Z. (2022). Synovial calprotectin for the diagnosis of periprosthetic joint infection: A diagnostic meta-analysis. J. Orthop. Surg. Res..

[197] Abdelnasser M.K., Bakhet A., Hosni A., Kamal D.T., Osman O.B., Abdelhameed M.A., Moustafa M.M. (2025). Alpha defensin immunoassay is more effective for ruling out rather than diagnosing periprosthetic joint infection (PJI): A prospective cohort study. Arthroplasty.

[198] Wang H., Qin L., Wang J., Huang W. (2021). Synovial fluid IL-1β appears useful for the diagnosis of chronic periprosthetic joint infection. J. Orthop. Surg. Res..

[199] Chen M.-F., Chang C.-H., Yang L.-Y., Hsieh P.-H., Shih H.-N., Ueng S.W.N., Chang Y. (2019). Synovial fluid interleukin-16, interleukin-18, and CRELD2 as novel biomarkers of prosthetic joint infections. Bone Jt. Res..

[200] Zou Y., Yang Y., Yang J., Zhang Y., Zhao C., Qin L., Hu N. (2025). The Utility of Synovial Fluid Interleukin-10 in Diagnosing Chronic Periprosthetic Joint Infection: A Prospective Cohort Study. Infect. Drug Resist..

[201] Rızvanoglu İ.H., Sakarya B., Benlier N., Kökçü F. (2023). HMGB-1 Levels in Painful Knee Arthroplasty: Is it Possible to Distingue Periprosthetic Joint Infection and Aseptic Loosening?. Indian J. Orthop..

[202] Lee J.A., Won D., Lee E.H., Lee S.-T., Park K.K., Shin S., Jeong S.J. (2025). Utilization of cell-free DNA metagenomic analysis for early detection and microbial identification in prosthetic joint infections: A prospective cohort study in Korea. Front. Cell. Infect. Microbiol..

[203] Cai Y., Liang J., Chen X., Zhang G., Jing Z., Zhang R., Lv L., Zhang W., Dang X. (2023). Synovial fluid neutrophil extracellular traps could improve the diagnosis of periprosthetic joint infection. Bone Jt. Res..

[204] Karlidag T., Bingol O., Keskin O.H., Durgal A., Yagbasan B., Ozdemir G. (2025). C-Reactive Protein to Albumin Ratio and Prognostic Nutrition Index as a Predictor of Periprosthetic Joint Infection and Early Postoperative Wound Complications in Patients Undergoing Primary Total Hip and Knee Arthroplasty. Diagnostics.

[205] Le D.H., Dayan J.M., Sarfraz A., Schwarzkopf R., Aggarwal V., Dayan A.J. (2025). The C-Reactive Protein Combination Ratios Outperform the Albumin-Globulin Ratio in Diagnosing Periprosthetic Joint Infection after Total Knee Arthroplasty. J. Arthroplast..

[206] Cao Q., Ning X., Fan P., Cheng T., Zhang Y., Cheng C., Dai Z. (2025). The role of ratio markers based on prealbumin in the diagnosis of periprosthetic joint infection. Front. Cell. Infect. Microbiol..

[207] Yu Y., Wen Y., Xia J., Dong G., Niu Y. (2025). Blood Cell Ratio Combinations for Diagnosing Periprosthetic Joint Infections: A Preliminary Study. Infect. Drug. Resist..

[208] Sabater-Martos M., Garcia O., Boadas L., Morata L., Soriano A., Martínez-Pastor J.C. (2025). Synovial glucose and serum-to-synovial-glucose ratio perform better than other biomarkers for the diagnosis of acute postoperative prosthetic knee infection. J. Bone JT Infect..

[209] Slovacek H., Khanna R., Poredos P., Poredos P., Jezovnik M., Hoppensteadt D., Fareed J., Hopkinson W. (2021). Interrelationship of MMP-9, Proteoglycan-4, and Inflammation in Osteoarthritis Patients Undergoing Total Hip Arthroplasty. Clin. Appl. Thromb./Hemost..

[210] Chen G.Y., Nuñez G. (2010). Sterile inflammation: Sensing and reacting to damage. Nat. Rev. Immunol..

[211] Jorch S.K., Kubes P. (2017). An emerging role for neutrophil extracellular traps in noninfectious disease. Nat. Med..

[212] Theocharis A.D., Skandalis S.S., Gialeli C., Karamanos N.K. (2016). Extracellular matrix structure. Adv. Drug Deliv. Rev..

[213] Rapp A.E., Zaucke F. (2023). Cartilage extracellular matrix-derived matrikines in osteoarthritis. Am. J. Physiol. Cell Physiol..

[214] Zou C., Jiang S., Li H., Zhao K., Cao D., Yang G. (2026). The NLRP3 Inflammasome: Mechanisms of Activation, Regulation, and Therapeutic Opportunities. MedComm.

[215] Migliorini F., Eschweiler J., Maffulli N., Schenker H., Baroncini A., Tingart M., Rath B. (2021). Autologous Matrix-Induced Chondrogenesis (AMIC) and Microfractures for Focal Chondral Defects of the Knee: A Medium-Term Comparative Study. Life.

[216] Cristea S., Predescu V., Dragosloveanu S., Cuculici S., Marandici N. (2016). Surgical Approaches for Total Knee Arthroplasty.

[217] Clesham K., Sheridan G.A., Greidanus N.V., Masri B.A., Garbuz D.S., Duncan C.P., Howard L.C. (2022). Minimally Invasive Intermuscular Approaches Versus Conventional Approaches in Total Hip Arthroplasty: A Systematic Review and Me-ta-Analysis. J. Arthroplast..

[218] Chan C.W., Qin L., Lee K.M., Zhang M., Cheng J.C., Leung K.S. (2006). Low intensity pulsed ultrasound accelerated bone re-modeling during consolidation stage of distraction osteogenesis. J. Orthop. Res..

[219] Cheung W.H., Wong R.M.Y., Choy V.M.H., Li M.C.M., Cheng K.Y.K., Chow S.K.H. (2021). Enhancement of osteoporotic fracture healing by vibration treatment: The role of osteocytes. Injury.

[220] Zhu R., Fang H., Wang J., Ge L., Zhang X., Aitken D., Cai G. (2024). Inflammation as a therapeutic target for osteoarthritis: A literature review of clinical trials. Clin. Rheumatol..

[221] Konstantinidis I., N. Papageorgiou S., Kyrgidis A., George Tzellos T., Kouvelas D. (2013). Effect of Non-Steroidal Anti-Inflammatory Drugs on Bone Turnover: An Evidence-Based Review. RRCT.

[222] Yu J., Wang B., Zhang F., Ren Z., Jiang F., Hamushan M., Li M., Guo G., Shen H. (2023). Single-cell transcriptome reveals Staphylococcus aureus modulating fibroblast differentiation in the bone-implant interface. Mol. Med..

[223] Nairne-Nagy J., Gupta R., Ramasamy B., Solomon L.B., Callary S.A. (2026). Machine Learning Based Diagnostic Models for Hip and Knee Prosthetic Joint Infection: A Systematic Review. J. Orthop. Res..

[224] Ghaffari A., Clasen P.D., Kappel A., Rasmussen J., Gurchiek R.D., Kold S., Rahbek O. (2025). Monitoring Gait Recovery After Total Knee Arthroplasty Using Wearable Sensors: Responsiveness of Gait Accelerations. J. Orthop. Res..

